# Participants, Usage, and Use Patterns of a Web-Based Intervention for the Prevention of Depression Within a Randomized Controlled Trial

**DOI:** 10.2196/jmir.2258

**Published:** 2013-08-20

**Authors:** Saskia M Kelders, Ernst T Bohlmeijer, Julia EWC Van Gemert-Pijnen

**Affiliations:** ^1^Department of Psychology, Health and TechnologyUniversity of TwenteEnschedeNetherlands; ^2^National Institute for Public Health and the EnvironmentBilthovenNetherlands; ^3^Center for eHealth Research and Disease ManagementDepartment of Psychology, Health and TechnologyUniversity of TwenteEnschedeNetherlands

**Keywords:** Web-based intervention, depression, use patterns, usage, adherence, design, engagement, attrition

## Abstract

**Background:**

Although Web-based interventions have been shown to be effective, they are not widely implemented in regular care. Nonadherence (ie, participants not following the intervention protocol) is an issue. By studying the way Web-based interventions are used and whether there are differences between adherers (ie, participants that started all 9 lessons) and nonadherers, more insight can be gained into the process of adherence.

**Objective:**

The aims of this study were to (1) describe the characteristics of participants and investigate their relationship with adherence, (2) investigate the utilization of the different features of the intervention and possible differences between adherers and nonadherers, and (3) identify what use patterns emerge and whether there are differences between adherers and nonadherers.

**Methods:**

Data were used from 206 participants that used the Web-based intervention Living to the full, a Web-based intervention for the prevention of depression employing both a fully automated and human-supported format. Demographic and baseline characteristics of participants were collected by using an online survey. Log data were collected within the Web-based intervention itself. Both quantitative and qualitative analyses were performed.

**Results:**

In all, 118 participants fully adhered to the intervention (ie, started all 9 lessons). Participants with an ethnicity other than Dutch were more often adherers (χ^2^_1_=5.5, *P*=.02), and nonadherers used the Internet more hours per day on average (F_1,203_=3.918, *P*=.049). A logistic regression showed that being female (OR 2.02, 95% CI 1.01-4.04; *P*=.046) and having a higher need for cognition (OR 1.02; 95% CI 1.00-1.05; *P*=.02) increased the odds of adhering to the intervention. Overall, participants logged in an average of 4 times per lesson, but adherers logged in significantly more times per lesson than nonadherers (F_1,204_=20.710; *P*<.001). For use patterns, we saw that early nonadherers seemed to use fewer sessions and spend less time than late nonadherers and adherers, and fewer sessions to complete the lesson than adherers. Furthermore, late nonadherers seemed to have a shorter total duration of sessions than adherers.

**Conclusions:**

By using log data combined with baseline characteristics of participants, we extracted valuable lessons for redesign of this intervention and the design of Web-based interventions in general. First, although characteristics of respondents can significantly predict adherence, their predictive value is small. Second, it is important to design Web-based interventions to foster adherence and usage of all features in an intervention.

**Trial Registration:**

Dutch Trial Register Number: NTR3007; http://www.trialregister.nl/trialreg/admin/rctview.asp?TC=3007 (Archived by WebCite at http://www.webcitation.org/6ILhI3rd8).

## Introduction

Depression has a high prevalence that poses a large burden on the health care system. Research shows that easily accessible interventions for indicated prevention (targeted at people at risk) are essential and can be cost-effective [1-3]. Web-based preventive interventions are seen as a possible format for these interventions and have been shown to be effective in reducing depressive symptoms [4-9].

Although Web-based interventions have been shown to be effective, Web-based interventions are still not widely implemented in regular care [10-13]. An issue is that not all Web-based interventions achieve the desired effects and many interventions struggle with the issue of nonadherence (ie, participants not following the intervention protocol) [10,11,14-16]. Although it is difficult to investigate a causal relationship of adherence with the effectiveness of Web-based interventions, studies have shown a relationship between adherence and increased effect of an intervention (ie, dose-effect relationship) [17,18].

In recent years, adherence has gained considerable attention. Eysenbach coined the phrase law of attrition [15], and since then there have been studies and reviews about the relationship between characteristics of participants and adherence (eg, [14,19]) and between characteristics of interventions and adherence [16,20,21]. In this study, we see adherence as following the intervention protocol (ie, using an intervention as intended by the developers), for example, completing all lessons. Although the earlier mentioned studies give insight into adherence as an outcome measure and give some recommendations how to plan for adherence, the process of adherence remains unclear. By studying the way Web-based interventions are used and whether there are differences between adherers and nonadherers, more insight can be gained into this process of adherence. Furthermore, it may be possible to extract design recommendations from this usage data and recommended use patterns for participants to increase the likelihood of adhering to the intervention.

There has been research into the usage and use patterns of Web-based interventions. Descriptive studies of freely accessible interventions have shown that they attract a considerable number of visitors, but that these visitors often interact with or access a fraction of what is possible in the intervention [22-30]. Furthermore, many studies have found that increased usage of particular features, such as completing assessments and self-monitoring, increased the effectiveness of the intervention [22,24,25,28-31]. However, insight into the way individuals use an intervention is still lacking. Particularly, insight into the patterns of use of individual participants may provide the foundation for design recommendations. Furthermore, this could lead to the formulation of usage patterns that are most likely to lead to adherence.

In addition to adherence as a process, there are still many questions regarding characteristics of respondents that may predict adherence. Studies have investigated the predictive value of demographics and disease-related measures (eg, [14,19]), but although significant predictors have been identified, the predictive value remains low and there has been a call for investigation of other characteristics that might prove to be more predictive [10,14,16,19]. The need for cognition and the need to belong might be such characteristics. The need for cognition refers to an individual’s tendency to engage in and enjoy effortful cognitive endeavors [32]. It is shown that people with a high need for cognition are more likely to engage in online activities that are more cognitively challenging [33]. As many Web-based interventions rely heavily on text and on cognitive effort to process information, it might be that individuals with a high need for cognition are more likely to adhere to a Web-based intervention. Furthermore, it has been proposed that higher levels of interactivity on health websites will lead to greater comprehension of the content, as a function of need for cognition [34], which predicts a relationship between need for cognition and adherence to Web-based interventions. The need to belong was introduced by Baumeister and Leary [35] and reflects that this desire to form interpersonal attachments is a fundamental motive that has important consequences for social functioning. Although the authors argue that the need to belong should be present to some degree in all humans in all cultures, they state that individual differences are to be expected [35]. In the context of Web-based interventions, which can be social in nature but are often something that is to be done alone, the need to belong may be a predictor for adherence (ie in Web-based interventions which are low in socialness); a higher need to belong may increase the likelihood for nonadherence.

This paper presents analyses of log data collected in a study into the adherence and effectiveness of a Web-based intervention for the prevention of depression, in which 118 of the 239 participants (49.4%) adhered to the intervention (ie, started all 9 lessons) [36]. The aims of the current study are (1) describe the characteristics of participants and investigate their relationship with adherence, (2) investigate the utilization of the different features of the intervention and possible differences between adherers and nonadherers, and (3) identify what use patterns emerge and whether there are differences between adherers and nonadherers.

## Methods

### Parent Study and Participants

The analyses described in this paper were performed on data collected in the parent study on the adherence and effectiveness of the Web-based intervention for the prevention of depression [36]. The parent study employed a fractional factorial experimental randomized controlled trial (RCT; NTR3007) design in which the influence of 5 components on adherence and clinical effectiveness of the Web-based intervention was studied using 8 intervention arms. This design entails that of each component, 2 levels were created and that each level of each component is present in half of the intervention arms. Participants were adults with mild to moderate depressive symptoms (>9 and <39 on the Center of Epidemiological Studies Depression scale; CES-D [37]) who completed our online screening procedure. For the current study, data from all participants who started the first lesson were used. Therefore, we used the data from 206 of 239 participants in the parent study. Detailed information on the participants, procedures, and design of the parent study can be found in Multimedia Appendix 1.

### Intervention

Following Van Gemert-Pijnen et al [10], we viewed a Web-based intervention as the whole of the content, system, and the service it provides. In this conceptualization, interaction is not content, system, or service, but rather it is an integral part of a Web-based intervention and, depending on the viewpoint, it can be regarded as belonging to either category. Subsequently, we will describe the intervention, Living to the Full, according to these categories. During the study, no changes were made to the Web-based intervention apart from fixing minor bugs.

### Content

The Web-based intervention called Living to the Full is based on Acceptance and Commitment Therapy (ACT) [38] and mindfulness [39,40] and has been published as a self-help book [41]. The intervention has been shown to be effective in reducing depressive and anxiety symptoms as a group course and as a self-help course with email support [42-44]. The Web-based intervention included 9 chronological lessons, each lesson consisting of psycho-educational material and exercises. These 9 lessons can be divided into 4 segments: part 1 (lesson 1) focuses on the view that forms the basis of the course; part 2 (lessons 2 and 3) focuses on becoming aware of coping strategies, their short term effectiveness, and lack of long term effectiveness; part 3 (lessons 4-6) focuses on learning the skills to accept suffering; and part 4 (lessons 7-9) focuses on the application of the learned lessons to daily life. The participants were asked to complete exercises both online and offline. Online exercises consisted of free-text questions, multiple-choice questions, and monitoring behavior in the Web-based diary, among others. Offline exercises consisted of practicing mindfulness, performing chosen actions, and practicing cognitive defusion, among others.

### System

**Figure 1:**
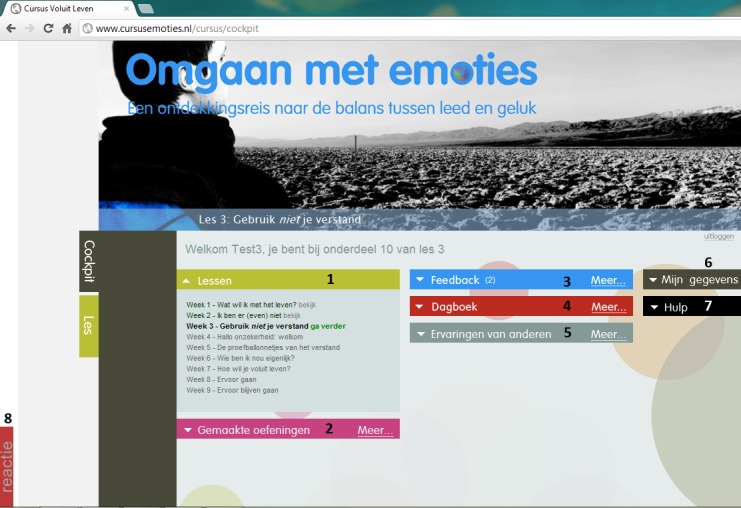
Personal home page of the Web-based intervention with the elements included for all participants.

The intervention was developed employing methods from the CeHRes Roadmap for eHealth development [10] and this process is described elsewhere [45]. When logging on to the Web-based intervention, participants start in their cockpit (Figure 1). From there, they can access all elements of the intervention. The elements that were included for all participants were (1) lessons, (2) overview of completed exercises, (3) feedback, (4) diary, (5) success stories, (6) my account, (7) help, and (8) a “react” button which allowed respondents to comment on the application.

### Service

For this study, the Web-based intervention was implemented in a research setting, namely at the University of Twente, the Netherlands. Participants could access the Web-based intervention at any time, from any place, free of charge. After finishing a lesson, participants could proceed to the next lesson after receiving feedback. This feedback was provided when a participant viewed all psycho-educational material and completed all exercises. Furthermore, feedback was sent at least 5 days after the participant started the lesson (see Multimedia Appendix 2 for the exact moment of feedback which differed for the levels of the support component). Participants were instructed to complete 1 lesson per week, but had 12 weeks in total to complete the 9 lessons. Participants were free to choose whether they worked through a lesson in 1 session or in multiple sessions. It was estimated that participants would spend an average of 3 hours per week on the intervention (online and offline activities combined).

### Interaction

Web-based interaction with the system consisted of doing online exercises, using multimedia content, and using personalized features. Interaction in the form of feedback messages (human or automated) was provided within the system as well. Furthermore, interaction with the system occurred through automated email messages that were sent to the participants’ email address to remind them to start, continue, or complete a lesson. For participants who signed up for short message service (SMS) coaching (see following paragraph), interaction also took place via their mobile phone. This interaction was 1-directional; there was no possibility to reply. Furthermore, all participants had the opportunity to contact the research staff by telephone, although this possibility was rarely used (approximately 5 phone calls during the intervention period in total).

### Intervention Components

#### Overview

Although the components of the intervention are not the focus of this study, this section will give a short overview of each of the levels of the components to be able to place the data presented in this study in its context. A detailed description can be found in Multimedia Appendix 2, and the foundations of these components can be found the parent study [36]. Each of the 8 intervention arms employed a different combination of levels of the intervention components. An overview of the composition of each of the intervention arms can be found in Multimedia Appendix 1.

#### Support

The source of support was either human or automated. To isolate the effect of the source of support, both conditions were designed as comparable as possible regarding length of feedback messages, tailored content, and presentation (including a photo of the counselor). To maintain the unique differences between human and automated support (increased possibility for interaction in human support and the increased possibility for timely feedback in automated support), participants in the human support condition had the opportunity to ask questions to their counselor, and participants in the automated support condition received 1 additional Web-based instant feedback message per lesson.

#### SMS Coaching

Participants in the condition that included SMS text messages had the opportunity to turn the SMS coach on. This SMS coach sent 3 predesigned text messages each week to a mobile phone number provided by the participant. The text messages were written by the researchers before the study started and the content was based on the results of the development study of the intervention [45]. Each week, 3 SMS text messages were sent containing motivational, mindfulness, and content-related information. All SMS text messages were presented in the text message tab of the application, independent of whether the SMS coach was turned on or off, but only for the participants in the condition that included text messages.

#### Experience Through Technology

The high experience condition contained additional multimedia and interactive material in the form of short movies, interactive exercises, and multimedia presentations of metaphors.

#### Tailoring of Success Stories

The intervention contained a success story for each lesson. For the high-tailored condition, each success story was tailored on 4 of the following aspects: gender, age, marital status, daily activity, most prominent symptom, and reason for participating in the Web-based intervention. The stories were tailored to a different combination of aspects each week and not on all aspects to maintain the credibility of the stories. In the low-tailored condition, a standard success story was presented each week.

#### Personalization

The high-personalization condition included personalized content that was adapted (the system shows the motto and picture selected by the participant; the system shows the most important values selected by the participant) and adaptable (possibility to create a personal top 5 of aspects from the course that the participant found most important).

### Data Collection and Analysis

Characteristics of participants were collected at baseline by using an online questionnaire. Depressive symptoms were measured with the CES-D (20 items, score 0-60; higher=more depressive symptoms [37,46]), anxiety symptoms were measured with the Hospital Anxiety and Depression Scale (HADS-A; 7 items, score 0-21; higher scores=more anxiety symptoms [47,48]). Need for cognition was measured using the Need for Cognition Short Form (18 items, score -54-54; higher scores=more need for cognition [32]). Need to belong was measured using the Need to Belong Scale (10 items, mean score 1-7; higher scores=more need to belong [49]). Internet usage was measured using 1 item (ie, “On average, for how many hours do you use the Internet per day?”). Internet experience was measured by using 10 items of the following format: “Do you ever use the following Internet applications?” The 10 items focused on the usage of search engines, webmail, online shopping, online banking, online communities, photo and video websites, (micro)blogs, chat, radio or music websites, and online (health) courses. The score was attained by counting the number of items that were answered with at least once in a while (range 0-10).

Usage of the Web-based intervention was measured objectively by log files. From these log files, adherence could be extracted. Adherence was defined as a participant starting lesson 9, because the intervention is intended to be used during the 9 lessons.

The log files contained a record of actions taken by each participant with for each action the following information: unique participant identification number, action type, action specification, and time and day. The action types that were logged were log-in, log-out, start lesson, start mindfulness, download mindfulness, view success story, view feedback message, start video, turn on SMS coach, turn off SMS coach, and view text message. Action specifications were, for example, the name of the mindfulness exercise started or which text message was viewed.

Descriptive analyses of use patterns were performed on 20 arbitrarily selected participants; 5 early nonadherers (ie, did not start lesson 5), 5 late nonadherers (ie, started lesson 5 but did not start lesson 9), and 10 adherers. We divided the nonadherers into early and late nonadherers to explore whether there were differences between these groups. It may be that people who nonadhere early have different reasons for nonadhering than late nonadherers. These early reasons may be more general aspects that become clear at an early stage (eg, the content is not attractive to them or the format of the intervention does not match their expectations). Late reasons may be more related to the process of the intervention or to the motivation (eg, it is hard to spend enough time each week on the intervention). It may be that late nonadherers are more similar to adherers and are easier to persuade to become adherers, whereas for early nonadherers, the intervention may simply not be suitable. Although the reasons for early or late nonadherence cannot be derived from this research, the results can show whether late nonadherers are more similar to adherers regarding their usage of the intervention. Effort was made to ensure that selected participants had the same distribution of demographic characteristics and randomized group as the full sample. Furthermore, we only selected participants who did not start to nonadhere in lessons 2, 5, or 8 because these were the lessons we investigated and we wanted to avoid including patterns of participants who did not complete the lessons under investigation. See Multimedia Appendix 3 for an overview of demographics, randomized group, and lesson reached of these selected participants. Of these participants, we examined all actions in lesson 2 (all selected participants), lesson 5 (late nonadherers and adherers), and lesson 8 (adherers only) to identify emerging use patterns. We chose to examine these lessons because they reflect the 3 main segments of the content of the intervention and because we wanted to avoid the first and the last lesson for the expected nonregular use pattern in these lessons; we expect the participants to explore and get to know the application more in the first lesson and the last lesson is shorter (ie, less text and exercises) than the other lessons. Of each lesson and for each selected participant, we recorded all actions between the time they started the lesson under investigation and the time they started the following lesson. Moreover, the number of sessions (a log-in and following actions until a log-out action or a period of 30 minutes of inactivity was counted as 1 session) was derived, as well as the total duration of these sessions and the time between session. Furthermore, the number of sessions used to complete all exercises and content of the lesson were counted. We chose to do this analysis only for a small subsample of the data because the focus of this exploratory analysis was on pattern recognition related to use of features of the interventions. Furthermore, the choice was pragmatic because of the lack of software to analyze log files, all analyses were done by hand.

Statistical analyses were done using PASW 18 (Predictive Analytics Software; IBM, USA). Differences between adherers and nonadherers were investigated using 1-way analyses of variance (ANOVA) and chi-square tests (χ^2^). Logistic regression was used to assess whether baseline characteristics predicted adherence. Because of the exploratory nature of the logistic regression, all predictor variables were added at once, using the enter method.

## Results

### Participant Characteristics

Baseline demographics and outcome measures of the 206 participants who used the intervention are shown in Table 1. There were differences between adherers and nonadherers on ethnicity (participants with an ethnicity other than Dutch were more often adherers; χ^2^_1_=5.5, *P*=.02) and Internet usage (nonadherers used the Internet significantly more hours per day on average; *F*_1,203_=3.918, *P*=.049). Women were more often adherers, but this did not meet statistical significance (χ^2^_1_=3.7, *P*=.05). Nonadherers had a higher need to belong, but this also did not meet statistical significance (*F*_1,204_=3.133, *P*=.08).

### Adherence

**Figure 2:**
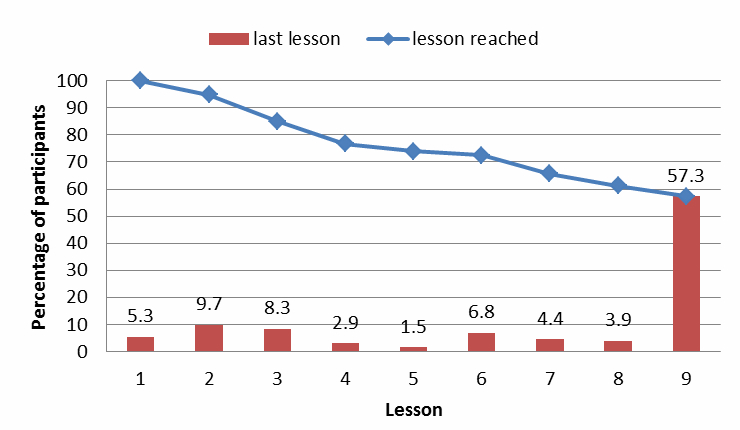
Graph of lessons completed against proportion of participants.

The average number of lessons started was 6.9 out of a possible 9, and 57% of the participants in this study completely adhered to the intervention (mode and median = 9 lessons). Figure 2 shows the percentage of participants who reached a certain lesson. From this figure, the largest group of nonadherers began to nonadhere in lesson 2, followed by lessons 3 and 6. Moreover, we can see that 26.2% (54/208) of participants were early nonadherers (ie, did not start lesson 5) and 16.6% (34/208) were late nonadherers (ie, started lesson 5 but did not start lesson 9).

To explore the possible predictive value of baseline characteristics for adherence (ie, starting all 9 lessons), we performed an exploratory logistic regression with all baseline characteristics showed in Table 1 entered as predictors. Table 2 shows that significant predictors in the model were gender and need for cognition, in which being female and having a higher need for cognition increased the odds of adhering to the intervention. A linear regression to predict the lesson reached by baseline characteristics yielded a significant model (χ^2^_12_=28.9, *P*=.004; Cox & Snell *R* ^*2*^=0.132, Nagelkerke *R* ^*2*^= 0.177), but no significant predictor variables (data not shown).

**Table 1:** Baseline demographics and outcome measures of all participants, and differences between adherers and nonadherers.

Participant characteristic	Total (N=206)	Adherers (n=118)	Nonadherers (n=88)	*P*
Age (years), mean (SD)	44.7 (12.5)	45.2 (12.6)	43.9 (12.3)	.47
Gender (women), n (%)	150 (72.8)	92 (78.0)	58 (65.9)	.05
**Ethnicity, n (%)**				.02
	Dutch	188 (91.3)	103 (87.3)	85 (96.6)
	Other	18 (8.7)	15 (12.7)	3 (3.4)
**Education, n (%)**				.51
	High	139 (67.5)	82 (69.5)	57 (64.8)
	Middle	53 (25.7)	30 (25.4)	23 (26.1)
	Low	14 (6.8)	6 (5.1)	8 (9.1)
**Marital status, n (%)**				.46
	Married	72 (35.0)	45 (38.1)	27 (30.7)
	Divorced	41 (19.9)	20 (16.9)	21 (23.9)
	Widowed	4 (1.9)	3 (2.5)	1 (1.1)
	Unmarried	89 (43.2)	50 (42.4)	39 (44.3)
**Daily activities, n (%)**				.15
	Paid job	131 (63.6)	69 (58.5)	62 (70.5)
	Student	16 (7.8)	9 (7.6)	7 (8.0)
	No job	59 (28.6)	40 (33.9)	19 (21.6)
CES-D, mean (SD)	24.9 (6.9)	24.5 (7.3)	25.4 (6.5)	.35
HADS-A , mean (SD)	9.7 (2.6)	9.4 (2.5)	10.0 (2.6)	.13

**Table 2:** Logistic regression baseline characteristics and adherence.

Included	B^a^ (SE)	*P*	OR (95% CI)
Constant	0.56 (1.82)	.76	
Age	–0.01 (0.02)	.65	0.99 (0.96-1.02)
Gender	0.70 (0.35)	.046	2.02 (1.01-4.04)
Ethnicity	1.29 (0.70)	.07	3.63 (0.92-14.26)
Education	0.30 (0.26)	.25	1.35 (0.81-2.24)
Marital status	–0.09 (0.14)	.53	0.92 (0.70-1.20)
Daily activities	0.35 (0.19)	.08	1.41 (0.97-2.06)
CES-D	–0.01 (0.02)	.71	0.99 (0.95-1.04)
HADS-A	–0.12 (0.07)	.07	0.89 (0.78-1.01)
Need for Cognition	0.02 (0.01)	.02	1.02 (1.00-1.05)
Need to Belong	–0.33 (0.27)	.22	0.72 (0.43-1.21)
Internet usage	–0.16 (0.09)	.06	0.85 (0.72-1.01)
Internet experience	–0.05 (0.11)	.64	0.95 (0.77-1.18)

^a^B: unstandardized coefficient.

### Usage

From the log files, the number of times each participant performed an action in the Web-based application was extracted (Table 3). Overall, participants logged in an average of 4 times per lesson, but adherers logged in significantly more times per lesson started than nonadherers (*F*_1,204_=20.710; *P*<.001). Other differences were that adherers downloaded a higher percentage of possible unique mindfulness exercises than nonadherers (*F*_1,204_=5.888; *P*=.02) and that adherers in the condition that included SMS coaching viewed a larger percentage of the possible text messages than nonadherers in that condition (*F*_1,103_=7.668; *P*=.007). To explore whether intervention components influenced the frequency of user actions, we compared the percentage of unique success stories that were viewed between participants in the condition with high- and low-tailored success stories and found that there was no significant difference. However, there was a difference between the total number of unique feedback messages viewed between the conditions with human and automated support (whole group: human support 10.7 unique messages viewed; automated support 5.9 unique messages viewed; *F*_1,204_=37.322, *P*<.001) and between the conditions on the number of unique messages viewed per lesson for adherers as well as for nonadherers (adherers: human support 1.7 per lesson, automated support 0.9 per lesson, *F*_1,116_=93.604, *P*<.001; nonadherers: human support 1.1 per lesson, automated support 0.6 per lesson, *F*_1,86_=23.860, *P*<.001).

### Use Patterns

To examine in more detail the way participants interacted with the system during the lessons, the use patterns of 20 participants (5 early nonadherers, 5 late nonadherers, and 10 adherers) on lesson 2 (all selected participants), lesson 5 (late nonadherers and adherers), and lesson 8 (adherers only) were investigated. Multimedia Appendix 4 presents all actions per participant per lesson, organized into sessions. Furthermore, Multimedia Appendix 4 presents the duration of each session, the time in between sessions, and an overview of the total duration of sessions and time between sessions per participant per lesson. A summary of this information for early nonadherers, late nonadherers, and adherers is presented in Table 4. From this table we can see that there seem to be differences between the use patterns of the 3 groups. First, early nonadherers used less sessions and spent less time than late nonadherers and adherers, and used less sessions to complete the lesson than adherers. Second, late nonadherers had a shorter total duration of the sessions than adherers, with the difference being more pronounced in lesson 5. Finally, adherers used less sessions (total and to complete a lesson) in the later lessons, but there was no visible trend for the duration of sessions and time between sessions, although they were a higher for lesson 5. When looking at the data in Multimedia Appendix 4, we observed some notable patterns:

1. There are many sessions that involve only a log-in and a log-out action, with less than a minute in between.
2. Adherers start the later lessons with a very short first session.
3. Many feedback messages are not read the first session after they are available.
4. There are many log-in actions shortly after another action.

**Table 3:** User actions of adherers and nonadherers.

User actions		Adherers (n=118)	Nonadherers (n=88)	Total (N=206)
**Log-in,** ^a^ **mean (SD)**				
	Total	40.2 (19.8)	14.4 (13.6)	29.1 (21.6)
	Per lesson	4.5 (2.2)	3.2 (1.5)	3.9 (2.0)
**Feedback messages viewed, mean (SD)**				
	Total	22.9 (17.6)	6.1 (7.8)	15.7 (16.5)
	Unique messages	12.0 (5.2)	3.8 (3.7)	8.5 (6.1)
	Unique messages per lesson	1.3 (0.6)	0.8 (0.6)	1.1 (0.6)
**Mindfulness exercises**				
	Total started, mean (SD)	7.8 (5.6)	3.6 (3.3)	6.0 (5.2)
	Unique started, mean (SD) %^b^	3.6 (1.4) 72.0%	2.1 (1.3) 74.3%	2.9 (1.6) 73.0%
	Unique downloaded, mean (SD) %^b^	2.6 (2.1) 51.5%	1.1 (1.3) 37.7%	1.9 (1.9) 45.6%
	Unique used, mean (SD) %^b^	4.4 (1.0) 87.6%	2.3 (1.3) 81.6%	3.5 (1.5) 85.0%
**Success stories viewed**				
	Total, mean (SD)	8.8 (7.5)	3.5 (3.5)	6.5 (6.7)
	Unique, mean (SD) %^b^	5.2 (2.8) 57.3%	2.4 (1.9) 61.4%	4.0 (2.8) 59.1%
**Text message coaching** ^c^				
	Participants that turned text message coaching on, n^d^	19	7	26
	Lessons turned on, mean (SD)^e^	7.9 (2.6)	2.4 (1.7)	6.5 (3.4)
	Total messages viewed, mean (SD)	14.3 (20.0)	2.4 (3.7)	9.6 (16.7)
	Unique messages viewed, mean (SD) %^b^	8.4 (8.9) 31.0%	1.8 (2.8) 14.9%	5.8 (7.8) 24.6%
**Movies viewed** ^f^				
	Total, mean (SD)	5.4 (6.1)	2.0 (3.8)	3.9 (5.5)
	Unique, mean (SD) %^b^	3.5 (3.4) 38.6%	1.3 (2.3) 25.5%	2.5 (3.2) 32.9%

^a^Log-ins within 30 minutes of the previous log-in were not counted to make the log-ins reflect the number of sessions more accurately; Log-ins per started lesson: number of log-ins divided by the number of the last lesson started.

^b^% = unique actions/possible actions. For adherers, the number of possible actions is the total number of available actions of that kind in the whole intervention. For nonadherers, the number of possible actions is the total number of available actions in all lessons that the participant started.

^c^Only for participants in the condition that included text message coaching; n=105; adherers n=63; nonadherers n=42.

^d^The number of participants that turned the text message coach on at least 1 time.

^e^The number of lessons the text message coach was turned on for the participants that turned the text message coach on at least 1 time.

^f^ Only for participants in the high experience condition; n=116; adherers n=65; nonadherers n=51.

**Table 4:** Mean number of sessions and duration for early nonadherers (n=5), late nonadherers (n=5), and adherers (n=10).

Variable	Early nonadherers, mean (SD)	Late nonadherers, mean (SD)		Adherers, mean (SD)		
Lesson	2	2	5	2	5	8
Total sessions	2.8 (1.6)	4.4 (1.5)	4.0 (1.6)	5.5 (2.6)	4.3 (1.3)	4.0 (1.9)
Sessions to complete lesson	1.8 (0.8)	2.0 (1.2)	2.8 (1.6)	3.5 (2.0)	2.8 (0.9)	1.9 (0.9)
Total duration of session (min)	36.2 (44.8)	64.0 (45.2)	38.8 (33.3)	101.9 (55.6)	125.6 (99.8)	114.0 (110.4)
Time in between sessions (days)	6.7 (4.1)	10.0 (4.1)	10.8 (1.8)	7.7 (1.7)	10.8 (6.1)	9.6 (5.2)

## Discussion

### Principal Results

The aims of this study were to (1) describe the characteristics of participants and investigate their relationship with adherence, (2) investigate the utilization of the different features of the intervention and possible differences between adherers and nonadherers, and (3) identify what use patterns emerge and whether there are differences between adherers and nonadherers.

The participants in this study were primarily Dutch females with a higher education level and a paid job. This group is similar to the group reached by many Web-based or eHealth interventions (eg, [15,19,22]), and this was the expected group which we took into account in the development process. When looking at differences between adherers and nonadherers, we see that although we reached only a small percentage of participants with an ethnicity other than Dutch; these participants were more often adherers. Others have stressed the importance and challenge of reaching people with non-Dutch ethnicity [13]. This study shows that if we can succeed in reaching this population, it may be easier to keep them engaged with a Web-based intervention, but this needs further research. Furthermore, nonadherers generally used the Internet for more hours per day than adherers. This finding is similar to other studies [19,50] and is something that deserves more research. One possible explanation is that people who differ in their amount of Internet use, also differ in the expectations they have of Web-based systems and, in this case, Web-based interventions. It may be that this Web-based intervention does not completely fit the mental model of a Web application of regular Internet users; the Web-based intervention, for example, may require more intense use as opposed to browsing where information is screened and many pages are viewed in a short amount of time. Our logistic regression model to predict adherence from characteristics of participants had relatively low predictive power (Nagelkerke *R* ^*2*^=0.177) in which only being female and having a higher need for cognition increased the odds of adhering to the intervention. The finding that women are more likely to adhere was mirrored in the finding that more women were adherers versus nonadherers (although this was statistically nonsignificant) and may reflect our choice to include more women as participants in the development process. Moreover, it strengthens the assumption that it is important to take the target group into account. If we intend to reach and engage men more, we should redesign the intervention using their input. The second significant predictor was the need for cognition, which supports our hypothesis that a higher need for cognition may be beneficiary for completing a Web-based intervention that relies substantially on text and on cognitive effort to process information. This implies that if an intervention is not only aimed at participants with a high need for cognition, attention should be paid to make the intervention more suitable for participants with a lower need for cognition. Although this model and other studies [14,19,51] show that individual differences play a role in adherence, the predictive value of the characteristics we measured is still small. A different approach has been used in the field of persuasive technology, where tailoring persuasive messages to personality traits has been shown to be effective [52]. Furthermore, in this field the question why certain individuals are persuaded and others are not has been investigated from a more practical view: by generating an individual persuasion profile from data on actual behavior, the most effective strategy to persuade this individual can be deduced and employed [53]. From there, one can theorize where these persuasion profiles come from and whether they can be measured in advance. This might be a practical way to tackle this issue in the field of Web-based interventions and eHealth.

Overall, of the 206 participants that used the application, 118 participants adhered to the intervention. Although we included the percentage of adherers by using these numbers, it should be noted that we only report on participants that started lesson 1. The true adherence derived from all participants is 49.4% (118/239) [36].This percentage is in-line with the average adherence found in a systematic review [16]. The data showed that most of the participants who did not adhere to the intervention, started to nonadhere during the first 3 lessons (55%, 48/88 participants that started the first lesson, but did not adhere to the intervention). This might reflect the content of the intervention, in which the first lesson focuses on whether the participants are really open for the therapy and the next 2 lessons focus on becoming aware that the coping strategies they use are not effective. This can be very confronting and may, therefore, explain the high nonadherence in these lessons. Interestingly, there is also a fairly large group of participants that start to nonadhere during lesson 6. This lesson is the last lesson in the segment on learning new skills to accept suffering, and this particular lesson focuses on the observing self. Counselors who have given this course know that this is hard lesson for many participants, which may explain the large group of nonadherers in this lesson. For the redesign of this Web-based intervention, this finding indicates that this might be a moment when the intervention should provide extra motivation, for example, through more interaction or simply by acknowledging that it is known that this is a hard moment to stick with the program. According to the supportive accountability model, human support may increase adherence at these times by providing the right amount (tailored to the individual) of support [54].

Our results on the usage of the different features mirror the results of studies on the usage of freely available Web-based interventions in that participants do not use all the features that they can use [22-30]. It seems that features that are an integral part of the therapy (eg, the mindfulness exercises in this study) are used more than additional features (success stories, text message coaching, and movies). This is something to keep in mind when designing or redesigning Web-based interventions: be aware that not all features in an application will be used and try to integrate features into the intervention instead of adding them onto the intervention. The success stories, for example, could be integrated more into the intervention by inserting them into each lesson. This is in-line with a recommendation from Krukowski et al [30] to encourage or more prominently feature certain features to increase usage and, ideally, lead to improved outcomes.

Significant differences between the user actions of adherers and nonadherers (ie, adherers showed more log-ons per lesson, downloaded more mindfulness exercises, and viewed more text messages than nonadherers) indicate that adherers not only have more endurance regarding usage during the full duration of the intervention, but are also more engaged with the intervention compared to nonadherers. This mirrors the finding that adherers show more involvement with the intervention [36]. This higher engagement within a lesson may be beneficial for the effectiveness of the intervention because more exposure to a Web-based intervention has been shown to increase the effectiveness (eg, [17,30]). For this specific intervention, completing a lesson in more than 1 log-in was recommended because the content requires repeated practice (eg, mindfulness and diary exercises) and time to reflect on the content before completing exercises. However, a participant could complete a full lesson in 1 log-in.

The average number of feedback messages viewed per lesson was below 1 for adherers and nonadherers, which shows that not all feedback messages have been viewed. Receiving feedback was the most wanted and expected feature of a Web-based intervention according to the participants in our development study, and providing support has been shown to have a positive effect on the effectiveness of Web-based interventions [4]. This makes the finding that not all feedback messages have been viewed even more striking. An explanation may be that this feature that was thought to be integral to the treatment by the developers was implemented in a way that did not reflect this integral nature; feedback messages were presented in a different section of the system than the lessons (the main part of the therapy) and participants could proceed to the next lesson without viewing the feedback message. Additionally, participants in the human support condition viewed more feedback messages than participants in the automated support condition. This finding is not surprising because the automated support condition included only 1 message per lesson, whereas the human support condition included the possibility to ask questions and request more feedback. Interestingly, the study into adherence and effectiveness of this intervention [36] did not show a significant difference in effectiveness at follow-up between these conditions, even though a counselor gave the feedback and more feedback messages were given (as shown in this study). These 2 findings show a need to further investigate the role of support and feedback in Web-based interventions.

Our analyses of the use patterns of 20 participants over 3 different lessons, provided us with useful insights. This more qualitative analysis confirmed our quantitative results on user actions: adherers are overall more engaged, they use more sessions, and spend more time with the intervention. Moreover, the analyses of the use patterns show us that there may be a difference between early nonadherers, late nonadherers, and adherers, in which late nonadherers are more similar to adherers in the number of sessions, but have a shorter duration of sessions, which is more similar to early nonadherers. This seems to fit our hypothesis that there is a difference between early and late reasons for nonadherence. Late nonadherers may be more similar to adherers and they may be easier to persuade to become adherers, whereas the intervention may simply not be suitable for early nonadherers. This should be investigated in future research.

By identifying differences between adherers and nonadherers, it becomes possible to screen for these wrong patterns and identify participants that are at risk to become nonadherers. This provides the opportunity to intervene, for example, by notifying these participants that they have a use pattern that increases the likelihood for nonadherence and suggesting a more appropriate use pattern. This combination of monitoring and self-monitoring of behavior and providing suggestions for different behavior are thought to be persuasive strategies for behavior change [55,56]. A different way to intervene may be to provide the participants with more or different interaction to increase adherence and effectiveness [54]. Although the current research provides a way to intervene, the specific action that is needed at that time for a specific participant should be the focus of future research.

Our in-depth analyses of the use patterns presented in Multimedia Appendix 4, yielded notable patterns that are useful for the redesign of this specific intervention. For example, the frequent log-in–log-out actions with no user action in between might be behavior of participants who were waiting for feedback. This hypothesis is supported by the finding that this pattern often occurs after participants have completed a lesson, but have not received feedback. A redesign option is to provide a prominent feature “when will I get my feedback?” where a timer can be shown with the expected time of feedback. This feature can then also be used to direct the participants to the features that they have not used at that time, to support participants to employ all the features to benefit most from the intervention. We saw that many adherers start the later lessons with a very short first session. This reflects the set-up of the intervention, in which the next lesson is only available after the participants complete the current lesson and a certain time since the start of the lesson has passed. This timer is started as soon as the lesson is started, so this first short session might be done to start the timer. The finding that many feedback messages are not read the first time they become available reflects the earlier finding that not all feedback messages are being read and might be improved by making it clearer that there is a new feedback message. A known bug in the application that has not been fixed is that a participant is logged out of the application when using the back button of the browser. This bug is a likely explanation of the many log-in actions shortly after another.

For this study, we used the log data of the Web-based intervention itself. This allowed us to identify actions of specific participants and relate them to whether the participant adhered to the intervention or not. Other studies have advocated the use of Google analytics, for example [57], but although this provides valuable information on a general level, it is not possible to identify specific participants, which diminishes the value of those methods for Web-based interventions that are intended to be used on multiple occasions. However, when developing a Web-based intervention, it is important to specify which information is important to be logged. For example, in our study, sessions were not logged as such, which meant that this had to be done manually, which is a tedious exercise. Furthermore, we manually wrote out all sessions for the selected participants in the selected lessons. Although this method provided valuable information, it is not feasible to do this for all participants for all lessons, which entails that analyses are done on a subset of the data. More advanced methods are needed to make use of all information that is collected. One such approach might be found in the use of Markov chains as used by Tian et al [58], although this might be less feasible for Web-based interventions that are intended to be used on more occasions. Another approach might be to employ pattern recognition methods from a machine learning perspective to see whether there are different patterns for adherers and nonadherers that can be automatically recognized or learned.

### Limitations

A limitation of this study is that we analyzed and interpreted log data without actively involving the participants. We did not ask participants why they used the intervention the way they used it. This information may have made it easier to interpret the data and to check whether our interpretation is correct. On the other hand, it is important to use objective log data and not to rely on subjective measures of how participants state that they used the intervention, because subjective data on usage are likely to be less accurate. Another limitation is the issue of generalizability. Our study used data from 1 intervention for the prevention of depression, which was used by primarily higher-educated Dutch women. Furthermore, we only investigated the use patterns of a small sample of these participants. The observed use patterns may be specific for this group using this intervention. However, many interventions, especially mental health interventions, have similar characteristics [16] and reach the same audience as stated earlier. Furthermore, the implications regarding designing for adherence, the limited predictive value of regular participant characteristics for adherence and the possibility to intervene based on screening of use patterns, seem to be broader than only for this intervention with this audience.

### Future Research

An interesting area for research can be found in a new way of analyzing the use patterns and investigating whether it is useful and feasible to intervene during the use of the intervention on the basis of the analyses of real-time use patterns. An earlier step might be to identify use patterns that are related to adherence and to design or redesign interventions in such a way to promote these use patterns. A different area of future research lies in the investigation of a more pragmatic way to identify participant characteristics that may influence or predict adherence, following the persuasion profiling approach [53]. Furthermore, our results indicate that the different content of lessons may need a different amount or mode of interaction. Here lies an interesting line of research: how can the content, system, service, and interaction of a Web-based intervention be attuned to one other to achieve the best match?

### Conclusion

In conclusion, we can say that using log data combined with baseline characteristics of participants of the intervention Living to the Full provided valuable lessons for redesign of this intervention and the design of Web-based interventions in general. First, although characteristics of respondents can significantly predict adherence, their predictive value is small. Therefore, we should look into other ways of classifying participants to make useful predictions about how individual difference may influence adherence. Second, it is important to design Web-based interventions to foster adherence and usage of all features in an intervention. A possibility for this is a smarter system that logs the current use pattern of a participant and intervenes when necessary, for example, by providing feedback or links to features that have not been accessed yet.

## Multimedia Appendix 1

Description of parent study.

## Multimedia Appendix 2

Detailed description of the five intervention factors.

## Multimedia Appendix 3

Characteristics of respondents for analyses of usage patterns.

## Multimedia Appendix 4

User actions, duration and time between sessions per participant per lesson.

## Multimedia Appendix 5

CONSORT-EHEALTH checklist V1.6.2 [59].

## Abbreviations

ACT: Acceptance and Commitment Therapy
ANOVA: analysis of variance
CES-D: Center of Epidemiological Studies Depression scale
HADS: Hospital Anxiety and Depression Scale
RCT: randomized controlled trial
SMS: short message service
