# Supplementary material for: Participants, Usage, and Use Patterns of a Web-Based Intervention for the Prevention of Depression Within a Randomized Controlled Trial
Source: J Med Internet Res. 2013 Aug 20;15(8):e172. doi: 10.2196/jmir.2258 (PMC3757912; doi:10.2196/jmir.2258)
Supplement: Supplementary file 1 [file jmir_v15i8e172_app1.pdf]

Multimedia Appendix 1 – Description of parent study [1]

Participants

Participants were recruited through advertisements in Dutch newspapers between February and March 2011. Inclusion criteria were an age of 18 year or older and mild to moderate depressive symptoms (>9 and <39 on the Center of Epidemiological Studies – depression scale; CES-D) .[2] People with severe depressive symptomatology and/or severe anxiety symptoms [more than 1 standard deviation above the population mean on the CES-D (cut-off score 39)[3] and/or on the Hospital Anxiety and Depression Scale – anxiety subscale (HADS-A;[4] cut-off score 15)][5] were excluded, because of the preventive nature of the intervention. Other exclusion criteria were: receiving psychological or psycho-pharmacological treatment for psychological complaints within the last 3 months; having less than 3 hours per week time to spend on the web-based intervention; poor Dutch language skills. The study was approved by an independent medical ethics committee (METIGG; no. NL33619.097.10) and recorded in the Dutch primary trial register for clinical trials (NTR3007).

Procedure

Interested people visited the study website. After viewing on screen information on the study and having the opportunity to download this information, informed consent was obtained from the participant through a checkbox and a pop-up screen to check whether they were sure to give informed consent. Participants then filled out an online screening questionnaire and were instantly informed whether they fulfilled the inclusion criteria. People who fulfilled the inclusion criteria were emailed a link to the online baseline questionnaire. A total of 239 respondents fulfilled the inclusion criteria, completed the online baseline questionnaire and were automatically randomized to one of eight intervention arms. All participants received an emailed link to the website of the web-based intervention on the same day (25 March). Respondents were not blinded to their randomized arm, but had no in-depth knowledge of the other arms. Participants received an emailed link to the online post intervention questionnaire three months after they could start the intervention. Six months after the start of the intervention period, participants received an emailed link to the online follow-up questionnaire. Participants received up to two automated email reminders when not filling out a questionnaire. Participants had no contact with the research staff, apart from the ability to ask questions via email or telephone.

Experimental Design

Based on the MOST method [6], a balanced fractional factorial design with 8 arms was chosen to screen for the effects of the five factors. Each level of each factor is present in half of the intervention arms. This design is called a Resolution III design and allowed for the estimation of all main effects (of the components), confounded by certain 2-way interactions. The design was intended to be balanced by having the same number of participants in each experimental arm. Due to a programming error, this was not achieved. The actual number of participants in each group is shown in Table 1.

Table 1 – Experimental groups of the fractional factorial design and the number of participants

Group	Support	Text messages	Experience	Tailoring	Personalization	Participants (n)
1	Automated	Yes	High	High	High	11
2	Automated	Yes	Low	Low	Low	43
3	Automated	No	High	Low	Low	36
4	Automated	No	Low	High	High	23
5	Human	Yes	High	Low	High	52
6	Human	Yes	Low	High	Low	19
7	Human	No	High	High	Low	35
8	Human	No	Low	Low	High	20

References

1. Kelders SM, Pots WTM, Bohlmeijer ET, Van Gemert-Pijnen JE. Persuasive technology, adherence and effect of a web-based intervention for the prevention of depression. Submitted.
2. Radloff LS. The CES-D scale: A self-report depression scale for research in the general population. Applied psychological measurement 1977;1(3):385-401. DOI: 10.1177/014662167700100306
3. Bouma J, Ranchor AV, Sanderman R, Van Sonderen E. Het meten van symptomen van depressie met de CES-D: Een handleiding [Measuring Symptoms of Depression with the CES-D, A Guide]: Noordelijk Centrum voor Gezondheidsvraagstukken, Rijksuniversiteit Groningen; 1995. ISBN: 9072156250
4. Zigmond AS, Snaith RP. The Hospital Anxiety and Depression Scale. Acta Psychiat Scand 1983;67(6):361-370. DOI: 10.1111/j.1600-0447.1983.tb09716.x
5. Olsson I, Mykletun A, Dahl AA. The hospital anxiety and depression rating scale: A cross-sectional study of psychometrics and case finding abilities in general practice. BMC Psychiatry 2005;5. DOI: 10.1186/1471-244x-5-46
6. Collins LM, Murphy SA, Strecher V. The multiphase optimization strategy (MOST) and the sequential multiple assignment randomized trial (SMART): new methods for more potent eHealth interventions. Am J Prev Med 2007 May;32(5 Suppl):S112-118. DOI: 10.1016/j.amepre.2007.01.022
