# Supplementary material for: Participants, Usage, and Use Patterns of a Web-Based Intervention for the Prevention of Depression Within a Randomized Controlled Trial
Source: J Med Internet Res. 2013 Aug 20;15(8):e172. doi: 10.2196/jmir.2258 (PMC3757912; doi:10.2196/jmir.2258)
Supplement: Supplementary file 2 [file jmir_v15i8e172_app2.pdf]

Multimedia Appendix 2 - Detailed description of the five intervention factors

Support

Participants randomized in the human support condition, received their weekly feedback from a human counselor. Participants randomized in the automated support condition, received weekly automatically generated feedback. The human counselors were four psychology Masters students of the University of Twente, who had received a 2-day workshop from an experienced clinical psychologist. The feedback messages were supervised by a clinical psychologist. The counselors were instructed to write a weekly feedback message containing the key learning points and goal of the completed lesson; the key exercises and feedback on at least the core exercise; feedback on the mindfulness exercise; and a preview of the following lesson. The automatically generated feedback contained the same elements, where the feedback on the core exercise and the mindfulness exercise was tailored based on the multiple choice responses of the participants to the question which was added to both exercises. An example question that was added after a core exercise was: 'Was writing down your 'bag of sorrow' confronting to you?". Feedback messages in both conditions were presented in the same manner (Figure MA2.1): under 'feedback' in the personal home screen, accompanied by a picture of the counselor. In the automated support condition, a picture of a clinical psychologist was placed who was not directly involved in the study. Participants were aware of whether their counselor was human or automated. Apart from the source of the feedback message, there were two differences in between the conditions. Participants in the human support condition had the opportunity to ask questions to their counselor. Questions were elicited when participants completed a lesson, but could also be asked at any time. Participants in the automated support condition, received one additional instant feedback message per lesson. This was a automatically generated message tailored to the multiple choice response of the participant on a different exercise than the core-exercise and was presented as a pop-up accompanied by the picture of the counselor.

Text messages

Participants in the condition that included text messages, had the opportunity to turn the SMS-coach on. This SMS-coach sent 3 pre-designed text messages each week to a mobile phone number provided by the participant. The timing of the text messages was different each week, but all messages were sent between 9AM and 9PM. Each week one message contained a motivational message (e.g. "Do you realize you have taken the first step to learn to 'live to the full'? Congratulations and keep going!"), one message contained a mindfulness trigger (e.g. "How mindful are you today?") and one message reflected on the content of that week (e.g. "Avoidance is like scratching an itch. It only works for a short time.") All text messages were presented in the 'text message' tab of the application, independent of whether the SMS-coach was turned on or off, but only for the participants in the condition that included text messages (Figure MA2.2).

Experience through technology

The high experience condition was different to the low experience condition in two aspects. In 8 of the 9 lessons, a short movie was added in which the writer of the course or an experiences clinical psychologist explains the key points of the lesson. The movie does not contain other information than the text, but the information is presented in a different way (Figure MA2.3). The second difference was that the high experience condition contained an interactive exercise or multimedia presentation of an exercise or metaphor in 7 of the 9 lessons (Figure MA2.4).

Tailoring of success stories

The intervention contained a success story for each of the lessons of the intervention that came available at the same time as the lessons. The participants accessed these stories from to cockpit , under 'experiences of others' and the stories were fictional, but based on the experiences of participants in an earlier study on the self-help book version of the intervention (Figure MA2.5). For the high tailored condition, each success story was tailored on 4 of the following aspects: gender, age, marital status, daily activity, most prominent symptom and the reason for participating in the web-based intervention. In the low tailored condition, each week a standard success story was presented. Hereby attention was paid to vary these stories on the aspects that were used for tailoring in the high tailored condition.

Personalization

Independent of condition, all respondents were addressed with their (reported) first name when logging on to the intervention in a welcome message (e.g. Welcome Saskia, you are at part 1 of lesson 4). Additionally, the high personalization condition (Figure MA2.6) showed the self-chosen picture and motto of the participant in the cockpit as soon as this was chosen in lesson 1; and showed the self-chosen most important values in the cockpit (from lesson 7 onwards). Furthermore, in this condition, participants had the opportunity to create their own 'top 5' of things from the course that they found most important. This top 5 was also showed in the cockpit. The low personalization condition did not provide these options.

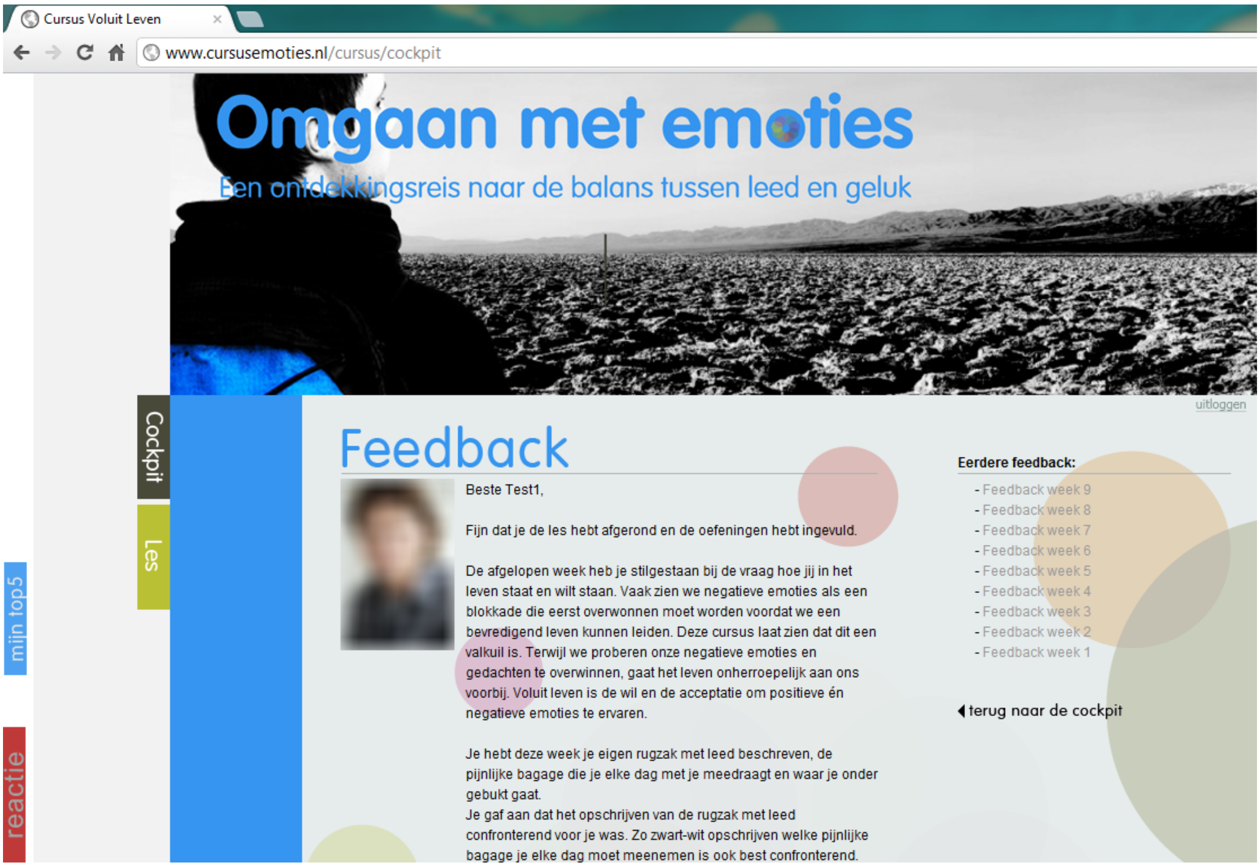

Figure MA2.1 – Feedback message in the automated support condition
Note: In this screenshot, the picture of the care-provider has been blurred for privacy.

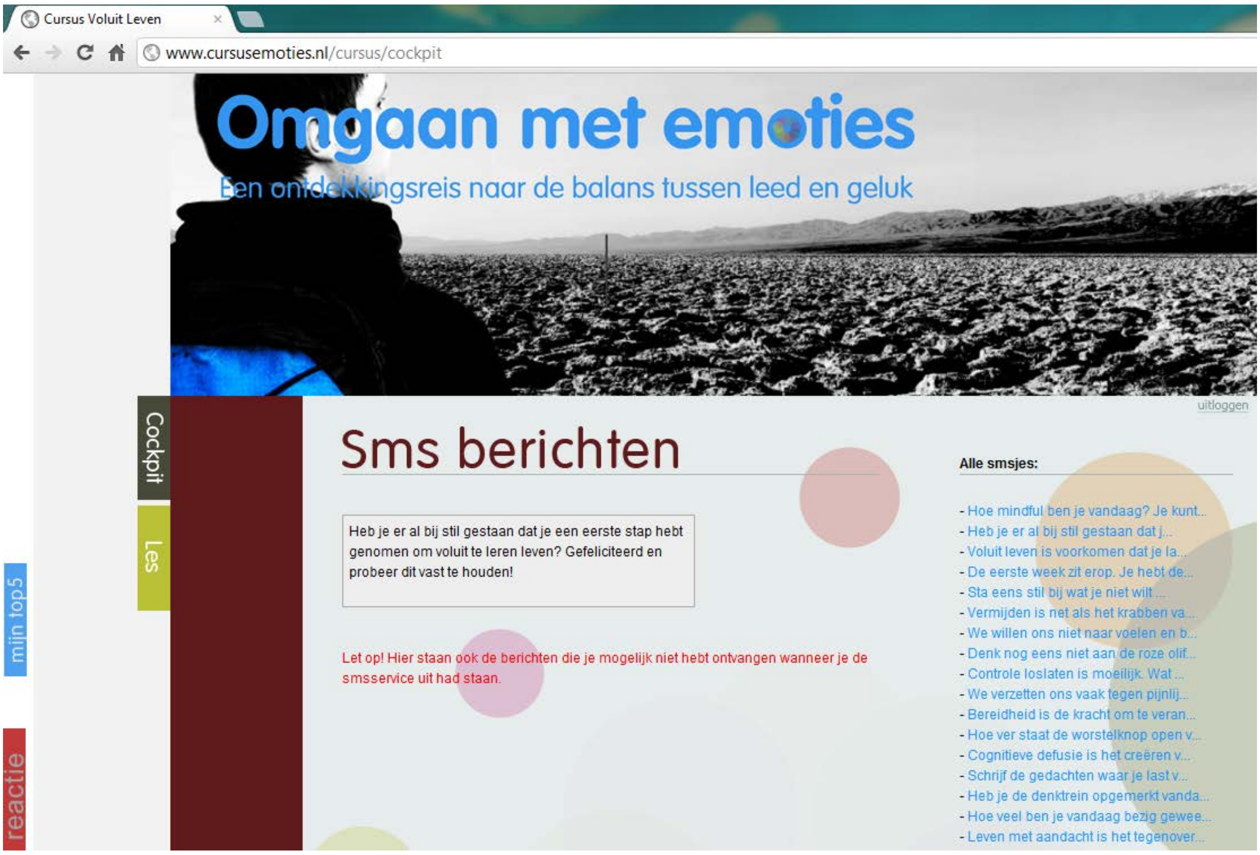

Figure MA2.2 – Text messages in the condition that included text messages

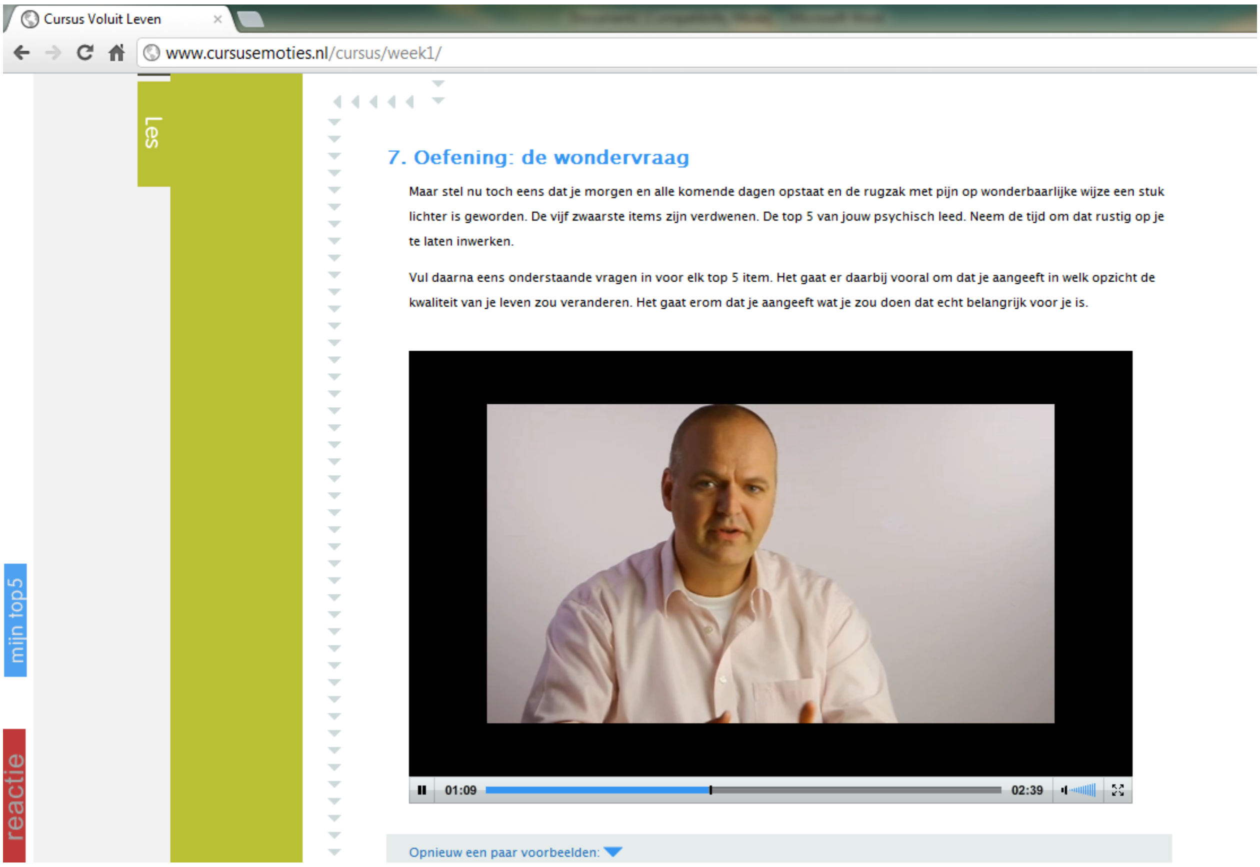

Figure MA2.3 – A movie in the high experience condition

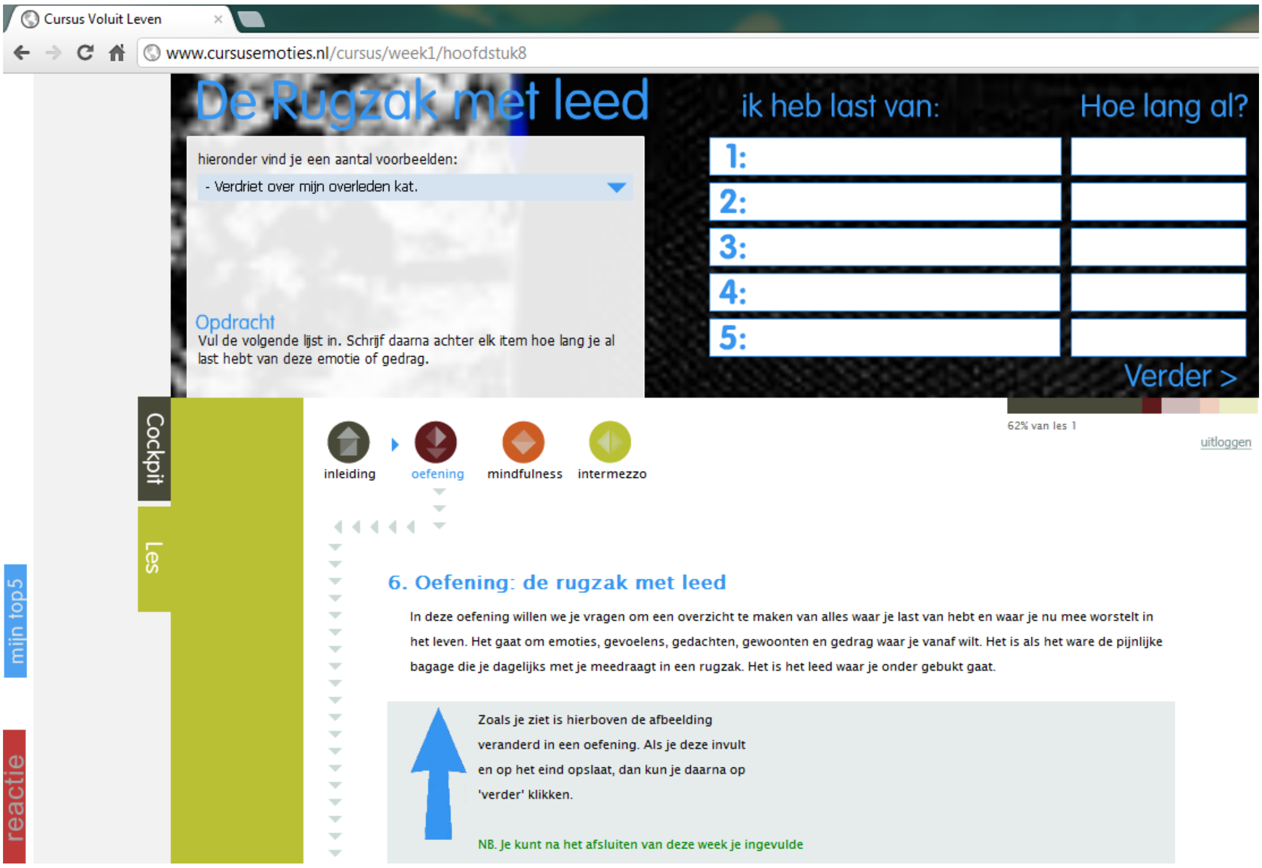

Figure MA2.4 – An interactive exercise in the high experience condition

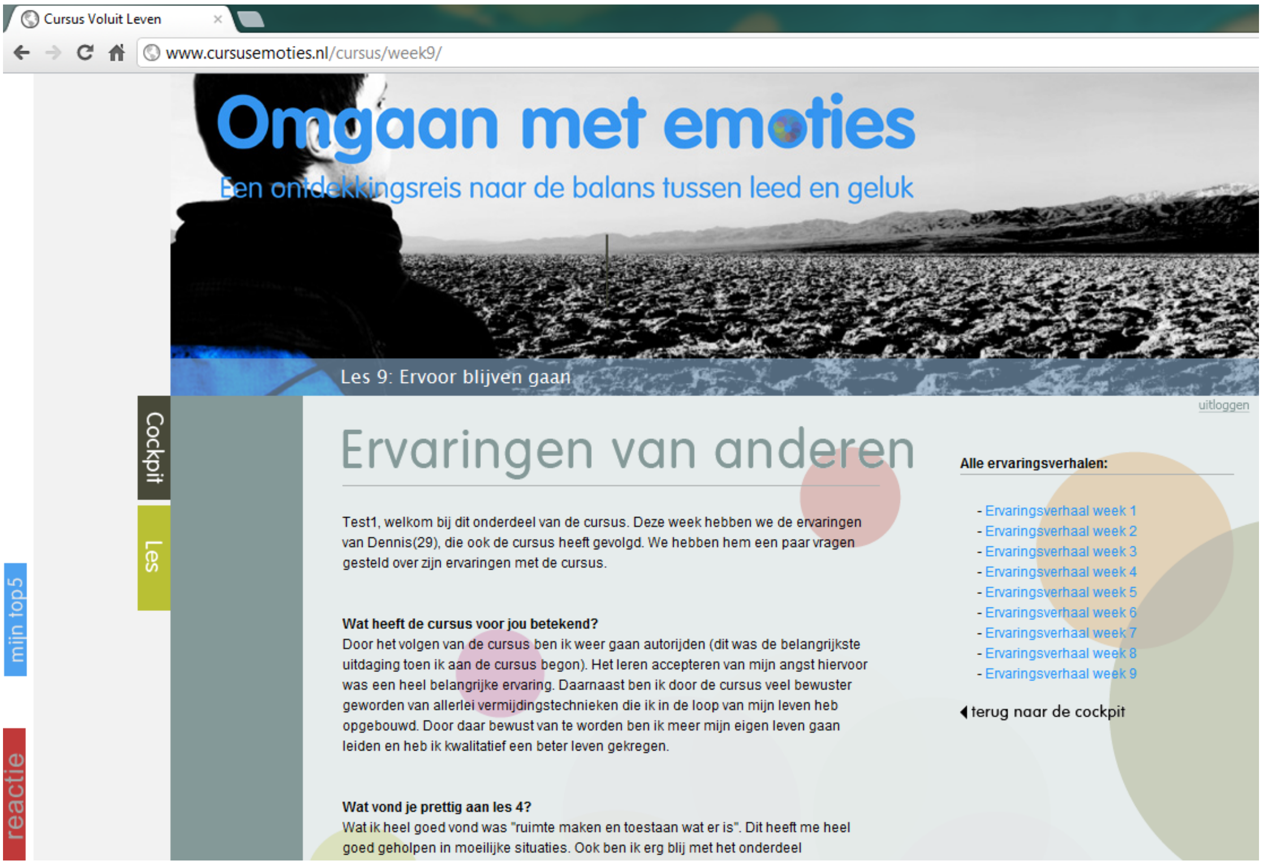

Figure MA2.5 – Example of a success story

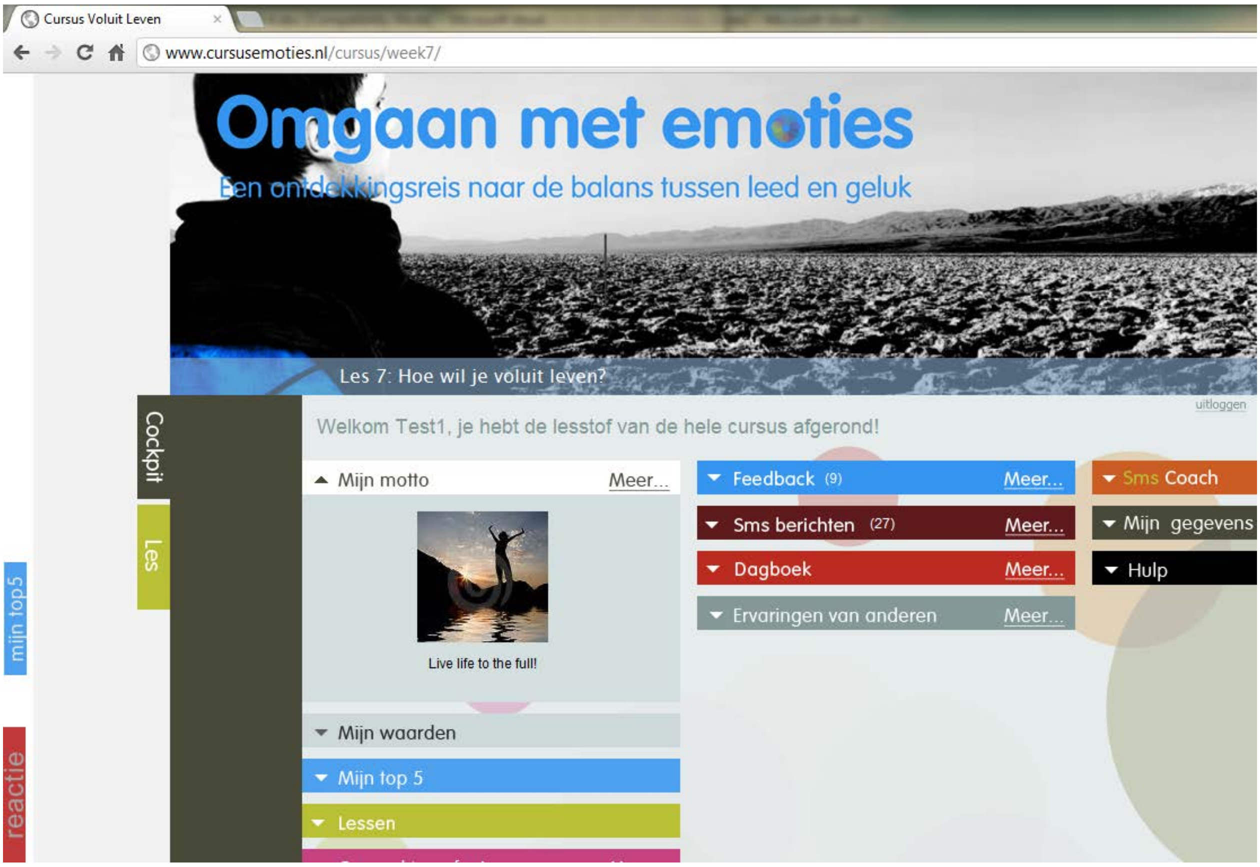

Figure MA2.6 – Cockpit of the high personalization condition
