# Supplementary material for: Participants, Usage, and Use Patterns of a Web-Based Intervention for the Prevention of Depression Within a Randomized Controlled Trial
Source: J Med Internet Res. 2013 Aug 20;15(8):e172. doi: 10.2196/jmir.2258 (PMC3757912; doi:10.2196/jmir.2258)
Supplement: Supplementary file 3 [file jmir_v15i8e172_app3.pdf]

Multimedia Appendix 3 – Characteristics of respondents for analyses of usage patterns

Table MA3.1 - Demographics, lesson reached and randomized group of arbitrarily selected participants for analyses of usage patters

No.	Lesson reached	Group	Gender	Age	Education	Internet usage (h/day)	Internet experience
1	3	7	female	23	high	2	6
2	3	5	male	46	medium	8	8
3	3	7	female	34	low	3	6
4	3	2	female	42	high	5	6
5	4	4	male	53	high	2	5
6	6	8	female	60	medium	2	7
7	6	7	male	44	high	8	6
8	6	4	male	56	high	1	6
9	7	3	female	66	high	4	3
10	7	1	female	40	medium	3	5
11	9	6	female	50	high	1.5	7
12	9	5	female	73	high	0.5	3
13	9	8	female	51	high	1	7
14	9	4	male	63	medium	4	6
15	9	5	female	44	high	0.5	2
16	9	8	male	60	high	2	4
17	9	4	male	35	high	5	7
18	9	5	female	36	medium	1	3
19	9	3	female	29	high	2	6
20	9	4	male	42	high	6	8
