# Supplementary material for: Participants, Usage, and Use Patterns of a Web-Based Intervention for the Prevention of Depression Within a Randomized Controlled Trial
Source: J Med Internet Res. 2013 Aug 20;15(8):e172. doi: 10.2196/jmir.2258 (PMC3757912; doi:10.2196/jmir.2258)
Supplement: Supplementary file 4 [file jmir_v15i8e172_app4.pdf]

Multimedia Appendix 4 - User actions, duration and time between sessions per participant per lesson

In the following tables, the user actions, duration and time between sessions of the selected participants are presented. There is are two separate tables for each lesson of each group of participants (early non-adherers, late non-adherers and adherers). The first table presents the user actions, divided into sessions. The second table presents the duration of each sessions in minutes and the time between sessions in days. Additionally, the last table presents the total number of sessions, the number of sessions taken to complete the lesson, the number of sessions before feedback was received, the total duration of sessions and the total time between sessions for each participant per lesson.

The following legend applies to all tables.

*: feedback received between sessions
+: finished lesson in the previous session
mf: mindfulness

Early dropouts, lesson 2

Table MA4.1 - User actions per participant per session

User	Session 1		Session 2		Session 3		Session 4		Session 5	
1	login; start lesson	*	login; continue lesson; multimedia; mf player 2; download mf 2; feedback 1877	+ *						
2	login; start lesson		login (3x); success story 2, login		login; continue lesson	+ *	login; feedback 2106			
3	login; start lesson, mf player 2; multimedia, logout	* +	login; feedback 3371							
4	start lesson; download mf 2		login; continue lesson; mf player 2 (2x); logout	+	login; feedback 1; success story 2		login	*	login; feedback 2	
5	start lesson; login; mf player 2; logout	+ *								

Table MA4.2 - Duration of sessions and time between sessions per participant

User	Session 1	Between	Session 2	Between	Session 3	Between	Session 4	Between	Session 5	Between
1	0 min	8 days (*)	22 min	(+ *)			-		-	
2	0 min	3 days	2 min	4 days	0 min	4 days (+*)	1 min		-	
3	22 min	8 days (+*)	1 min				-		-	
4	38 min	2 days	70 min	3 days (+)	6 min	1 day	0 min	12 hours (*)	1 min	
5	18 min	(+*)	-		-		-		-	

Late dropouts, lesson 2

Table MA4.3 - User actions per participant per session

User	Session 1		Session 2		Session 3		Session4		Session 5	
5	login; start lesson		login; continue lesson	+	login; success story 3; login	*	login; feedback 2146			
6	login; start lesson; multimedia, download mf 2, mf player 2, logout	+	login, logout		login, logout	*	login; feedback 2894; logout			
7	login; start lesson; login; continue lesson; logout	+	login; mf player 2; download mf 2; success story 3; logout	*	login; feedback 2					
8	login, start lesson; login		login; continue lesson; video 2; logout		login; video 2; continue lesson; logout		login; continue lesson; multimedia; mf player 2	+	login; video 2	
	**Session 6**		**Session 7**							
	login; success story 1, 2 (2x); feedback 1; video 2; mf player 2; logout	*	login; feedback 2							
	**Session 1**		**Session 2**		**Session 3**		**Session4**		**Session 5**	
9	login; start lesson; logout		login; feedback 1; continue lesson; download mf 2; sms 5; logout	+	login; feedback 1; logout	*	login feedback 2			

Table MA4.4 - Duration of sessions and time between sessions per participant

User	Session 1	Between	Session 2	Between	Session 3	Between	Session 4	Between	Session 5	Between
6	0 min	2 days	0 min	3 days (+)	1 min	2 days (*)	1 min			
7	35 min	7 days (+)	2 min	2 days	0 min	5 days (*)	36 min			
8	22 min	1 day (+)	17 min	6 days (*)	0 min					
9	2 min	1 day	15 min	2 days	31 min	6 hours	18 min	2 days (+)	1 min	1 day
	**Session 6**		**Session 7**							
	52 min	13 hours (*)	1 min							
	**Session 1**	**Between**	**Session 2**	**Between**	**Session 3**	**Between**	**Session 4**	**Between**	**Session 5**	**Between**
10	23 min	2 days	16 min	1 day (+)	46 min	12 days (*)	1 min			

Late dropouts, lesson 5

Table MA4.5 - User actions per participant per session

User	Session 1		Session 2		Session 3		Session 4		Session 5	
5	start lesson; mf player 5	+	login; feedback 2988	*	login; feedback 3355		login; feedback 3355			
6	start lesson; logout		login; continue lesson, logout		login; logout		login; continue lesson; mf player 5; logout		login; continue lesson; logout	+ *
	**Session 6**									
	login; feedback 4487; login									
**User**	**Session 1**		**Session 2**		**Session 3**		**Session 4**		**Session 5**	
7	start lesson; download mf 5 (2x); logout		login; success story 5, 6; continue lesson	+						
8	start lesson; video 5; logout		login (2x);		login; continue lesson; video 5; logout		login; continue lesson; mf player 5; logout	+ *	login; feedback 5	
9	start lesson; logout		login (2x); continue lesson; mf player 5; sms 13,14,15; logout	+	login; success story 1 and 6; logout	*				

Table MA4.6 - Duration of sessions and time between sessions per participant

User	Session 1	Between	Session 2	Between	Session 3	Between	Session 4	Between	Session 5	Between
6	12 min	5 days (+)	0 min	11 hours (*)	0 min	3 days	0 min			
7	0 min	41 min	7 min	6 days	0 min	1 day	8 min	2 days	5 min	4 days (+*)
	**Session 6**									
	33 min									
	**Session 1**	**Between**	**Session 2**	**Between**	**Session 3**	**Between**	**Session 4**	**Between**	**Session 5**	**Between**
8	17 min	11 days	1 min	+						
9	14 min	7 days	6 min	12 hours	52 min	1 day	19 min	1 day (+*)	0 min	
10	1 min	10 days	17 min	2 days (+)	2 min	*				

Adherers, lesson 2

Table MA4.7 - User actions per participant per session

User	Session 1		Session 2		Session 3		Session4		Session 5	
11	start lesson; logout		login; feedback 2674 and 2415; login; feedback 2674; logout; login		login; logout		login; continue lesson; download mf 2; mf player 2; logout	+ *	login; feedback 2978; logout	
	**Session 6**		**Session 7**		**Session 8**					
	login; success story 3; mf player 2; sms 5,2,5;	*	login; feedback 3123; logout		login; feedback 3123; mf player 1; login; mf player 2					
	**Session 1**		**Session 2**		**Session 3**		**Session4**		**Session 5**	
12	start lesson; video 2 (3x); mf player 2; login (4x)	+	login (2x); sms 6,5,4,3,2		login	*				
13	login; start lesson; logout		login; continue lesson; logout		login; mf player 1 (2x); download mf 1; logout		login; continue lesson; logout		login; feedback 1371 (2x); logout	+ *
	**Session 6**									
	login feedback 1802; 1387; logout									
	**Session 1**		**Session 2**		**Session 3**		**Session4**		**Session 5**	
14	login; start lesson; mf player 2; logout		login (2x); mf player 2; success story 1, 2, 3, 3; logout	+ *	login; feedback 1,2					
15	start lesson; feedback 2196; logout		login; continue lesson; sms 4; logout		login; continue lesson; logout		login; continue lesson; logout		login; download mf 2 (3x); login; download mf 2 (4x); logout; download mf 2 (5x)	+ *
	**Session 6**									
	login; download mf 2; multimedia; sms 5									
	**Session 1**		**Session 2**		**Session 3**		**Session4**		**Session 5**	
16	login; start lesson		login (2x); logout		login; continue lesson; logout		login; feedback 1427; logout		login; continue lesson; mf player 2 (2x); download mf 2;	+ *

									success story 3, 2; logout	
	**Session 6**									
	login; feedback 1427 (2x), 1814 (5); success story 3									
	**Session 1**		**Session 2**		**Session 3**		**Session4**		**Session 5**	
17	login; start lesson; logout		login; feedback 1; login; continue lesson; logout		login; continue lesson; download mf 2; logout	+ *				
18	start lesson; logout	*	login; continue lesson; multimedia; logout	+ *	login; feedback 2886; logout		login; feedback 2435; mf player 2 (2x)			
19	login; start lesson; logout		login; continue lesson; multimedia; logout		login; mf player 2 (2x)		login; download mf 2; logout		login; logout	
	**Session 6**		**Session 7**		**Session 8**		**Session 9**		**Session 10**	
	login; feedback 1; logout		login; continue lesson; multimedia; logout	+	login; multimedia		login; feedback 2; logout		login	
	**Session 11**									
	login; success story 2, 3	*								
	**Session 1**		**Session 2**		**Session 3**		**Session4**		**Session 5**	
20	login; start lesson; download mf 2; logout	+	login; logout		login; logout		login	*	login; feedback 2; logout	

Table MA4.8 - Duration of sessions and time between sessions per participant

User	Session 1	Between	Session 2	Between	Session 3	Between	Session 4	Between	Session 5	Between
11	6 min	26 min	37 min	1 day	1 min	1 day	1h 48 min	3 days (+*)	1 min	1 day
	**Session 6**		**Session 7**		**Session 8**					
	30 min	1 day (*)	2 min	2 days	8 min					
	**Session 1**	**Between**	**Session 2**	**Between**	**Session 3**	**Between**	**Session 4**	**Between**	**Session 5**	**Between**
12	1h 36 min	4 days (+)	3 min	1 day	0 min	*				
13	11 min	2 days	12 min	6 hours	26 min	3 days	8 min	3 hours	30 min	1 day (+*)
	**Session 6**									
	3 min									
	**Session 1**	**Between**	**Session 2**	**Between**	**Session 3**	**Between**	**Session 4**	**Between**	**Session 5**	**Between**
14	22 min	5 days	39 min	3 days (+*)	1 min					
15	27 min	1 day	29 min	1 day	12 min	3 days	1h 10 min	1 day	19 min	1 day (+*)
	**Session 6**									
	19 min									
	**Session 1**	**Between**	**Session 2**	**Between**	**Session 3**	**Between**	**Session 4**	**Between**	**Session 5**	**Between**
16	0 min	1 day	43 min	1 day	45 min	1 day	3 min	1 day	28 min	4 days (+*)
	**Session 6**									
	27 min									
	**Session 1**	**Between**	**Session 2**	**Between**	**Session 3**	**Between**	**Session 4**	**Between**	**Session 5**	**Between**
17	26 min	6 days	6 min	8 hours	13 min	+*				
18	8 min	5 days (*)	26 min	4 days (+*)	1 min	2 days	5 min			
19	15 min	4 hours	8 min	21 min	1 min	5 hours	1 min	1 day	46 min	2 hours
	**Session 6**		**Session 7**		**Session 8**		**Session 9**		**Session 10**	
	18 min	9 min	18 min	5 days (+)	8 min	1 day	2 min	10 hours	0 min	1 day
	**Session 11**									
	3 min	*								
	**Session 1**	**Between**	**Session 2**	**Between**	**Session 3**	**Between**	**Session 4**	**Between**	**Session 5**	**Between**
20	24 min	1 day (+)	9 min	2 days	14 min	3 days	0 min	1 day (*)	1 min	

Adherers, lesson 5

Table MA4.9 - User actions per participant per session

User	Session 1		Session 2		Session 3		Session 4		Session 5	
11	login; start lesson; logout		login; logout;		login; continue lesson		logout; login; mf player 5; download mf 5; success story 6; logout	+	login; sms 15; logout	
	**Session 6**		**Session 7**							
	login; logout	*	login; feedback 4390							
	**Session 1**		**Session 2**		**Session 3**		**Session 4**		**Session 5**	
12	login; start lesson; video 5; logout		login; continue lesson; mf player 5	+ *	login; feedback 2496, 4598; login (2x); feedback 4598, 4411, 4522, 4411, 3489; login (2x); feedback 4598		login; feedback 4598		login; feedback 4418 (sequence 3x); login; feedback 4598	
13	login; start lesson; logout		login; continue lesson; logout		login; continue lesson; mf player 5; logout		login; continue lesson; logout	+ *	login; feedback 2990; logout	
14	login; start lesson; logout		login; continue lesson; logout		login; continue lesson; download mf 5 (8x); mf player 5; logout; login; logout	+ *				
15	login; start lesson; feedback 3610, 3276, 3361, 3477, 3610; logout		login; continue lesson; logout		login (2x); download mf 5 (8x); logout; login; logout	+ *				
16	start lesson; feedback 2656 (2x); logout		login; continue lesson; mf player 5; download mf 2; logout; login; success story 6, 5; mf player 5; logout	+	login; feedback 2656	*	login; feedback 3335; logout			
17	login; start lesson; mf player 5 (2x); logout		login; feedback 4; continue lesson; download mf 5	+ *	login; feedback 5 (2x)					
18	start lesson; logout		login; continue lesson; download mf 5; success story 5, 6; logout	+	login; logout		login; logout	*	login; feedback 4445	

19	start lesson		login; continue lesson; mf player 5; download mf 5	+*	login; feedback 5; login					
20	login; start lesson; logout		login; logout		login; continue lesson; download mf 2 (2x); logout		login; continue lesson; logout	+*	login; feedback 5	

Table MA4.10 - Duration of sessions and time between sessions per participant

**User**	**Session 1**	**Between**	**Session 2**	**Between**	**Session 3**	**Between**	**Session 4**	**Between**	**Session 5**	**Between**
11	4 min	2 days	3h 38 min	18 min	0 min	3 hours	22 min	3 days (+)	5 min	1 day
	**Session 6**		**Session 7**							
	3 min	3 days (*)	0 min							
	**Session 1**	**Between**	**Session 2**	**Between**	**Session 3**	**Between**	**Session 4**	**Between**	**Session 5**	**Between**
12	20 min	3 days	14 min	5 days (+*)	28 min	3 days	1 min	3 days	3 min	
13	7 min	7 hours	12 min	2 hours	16 min	2 days	30 min	1 day (+*)	4 min	
14	1 min	5 days	3 min	2 days	35 min	+*				
15	18 min	2 days	39 min	2 days	1h 11 min	+*				
16	13 min	8 days	54 min	1 day (+)	34 min	3 days (*)	2h 58 min			
17	54 min	23 days	9 min	2 days (+*)	6 min					
18	3h 51 min	4 days	28 min	2 days (+)	5 min	4 days	1 min	2 days (*)	0 min	
19	0 min	4 days	48 min	6 days (+*)	1 min					
20	11 min	1 day	10 min	1 day	18 min	5 days	1 min	4 days (+*)	0 min	

Adherers, lesson 8

Table MA4.11 - User actions per participant per session

User	Session 1		Session 2		Session 3		Session4		Session 5	
11	Start lesson; download mf 8; logout		login; feedback 4688; continue lesson	+ *	login; feedback 4811					
12	start lesson; login; video 8 (4x); feedback (28x); login (4x); success story 9, 8; login (2x)	+	login (3x); feedback 2496; login (3x); video 3; login (5x);		login; logout		login (2x); download mf 5,6,8; login	*	login; download mf 1; login (3x)	
	**Session 6**									
	login; feedback 4892									
	**Session 1**		**Session 2**		**Session 3**		**Session4**		**Session 5**	
13	login; start lesson; success story 7, 8; logout		login; continue lesson; mf player 8; logout		login; continue lesson; feedback 3800; logout	+ *	login; feedback 4173; success story 7; logout	*	login; feedback 4052; logout	*
	**Session 6**		**Session 7**							
	login; feedback 4278, 4384; logout		login; feedback 4278, 4384							
	**Session 1**		**Session 2**		**Session 3**		**Session4**		**Session 5**	
14	login; start lesson; mf player 8; download mf 8 (8x)	+ *								
15	lesson start; mf player 8 (2x); logout	+	login (2x); sms 23, 23, 24; logout	*	login (2x); feedback 4707					
16	start lesson; logout		login; continue lesson; download mf 5; success story 9,8; logout	+	login; logout		login		login; feedback 4176; logout	*
	**Session 6**									
	login; feedback 4497									
	**Session 1**		**Session 2**		**Session 3**		**Session4**		**Session 5**	
17	login; start lesson; download mf 8; logout	+ *	login; feedback 6, 7, 8, 7							
18	start lesson; logout		login; continue lesson;		login; continue lesson; mf	+	login; logout	*		

			logout		player 8; download mf 8; sms (24x); logout					
19	login; start lesson; download mf 8, 5, 6		login		login; continue lesson; login	+	login		login	
20	start lesson; download mf 8; logout		login; continue lesson; logout	+	login (3x)	*				

Table MA4.12 - Duration of sessions and time between sessions per participant

**User**	**Session 1**	**Between**	**Session 2**	**Between**	**Session 3**	**Between**	**Session4**	**Between**	**Session 5**	**Between**
11	26 min	2 days	12 min	6 days (+*)	0 min					
12	1h 26 min	3 days (+)	48 min	2 days	1 min	5 hours	1h 51 min	1 day (*)	27 min	5 days
	**Session 6**									
	0 min									
	**Session 1**	**Between**	**Session 2**	**Between**	**Session 3**	**Between**	**Session4**	**Between**	**Session 5**	**Between**
13	24 min	3 days	16 min	2 days	25 min	6 days (+*)	4 min	1 day (*)	15 min	4 days (*)
	**Session 6**		**Session 7**							
	4 min	2 days	0 min							
	**Session 1**	**Between**	**Session 2**	**Between**	**Session 3**	**Between**	**Session4**	**Between**	**Session 5**	**Between**
14	13 min	+*								
15	1h 26 min	5 days (+)	42 min	7 days (*)	2h 33 min					
16	9 min	7 days	3h 23 min	2 days (+)	12 min	3 days	0 min	1 day	11 min	3 days (*)
	**Session 6**									
	0 min									
	**Session 1**	**Between**	**Session 2**	**Between**	**Session 3**	**Between**	**Session4**	**Between**	**Session 5**	**Between**
17	8 min	9 days (+*)	0 min							
18	1 min	1 day	8 min	3 days	2h 10 min	1 day (+)	0 min	*		
19	39 min	3 hours	0 min	7 days	2 min	5 hours (+)	0 min	1 day	0 min	
20	11 min	4 days	13 min	4 days (+)	0 min	*				

Table MA4.13 - Total number of sessions, number of sessions to complete the lesson, number of sessions before feedback was received, total duration of sessions and the total time between sessions for each participant per lesson

User	Lesson	Sessions			Duration of sessions (min)	Time between sessions (days)
		Total	Complete	Feedback		
1	2	2	2	1;2	22	8
2	2	4	3	3	3	11
3	2	2	1	1	23	8
4	2	5	2	4	115	6.5
5	2	1	1	1	18	0
6	2	4	2	3	2	7
	5	4	1	2	12	8.5
7	2	4	1	3	73	14
	5	6	5	5	53	11
8	2	3	1	2	39	7
	5	2	2	-	18	11
9	2	7	4	6	120	7
	5	5	4	4	91	9.5
10	2	4	2	3	86	15
	5	3	2	3	20	12
11	2	8	4	4;6	193	9
	5	7	4	6	252	9
	8	3	2	2	38	8
12	2	3	1	3	99	5
	5	5	2	2	66	14
	8	6	1	4	273	11.5
13	2	6	5	5	90	6.5
	5	5	4	4	69	3.5
	8	7	3	3;4;5	88	18
14	2	3	2	2	62	8
	5	3	3	3	39	7
	8	1	1	1	13	0
15	2	6	5	5	176	7
	5	3	3	3	128	4
	8	3	1	2	281	12
16	2	6	5	5	146	8
	5	4	2	3	279	12
	8	6	2	5	235	16
17	2	3	3	3	45	6.5
	5	3	2	2	69	25
	8	2	1	1	8	9
18	2	4	2	1;2	40	11
	5	5	2	4	265	12
	8	4	3	4	139	5
19	2	11	7	11	120	9
	5	3	2	2	49	10
	8	5	3	-	41	8.5
20	2	5	1	4	48	7
	5	5	4	4	40	11
	8	3	2	3	24	8
