# Supplementary material for: Participants, Usage, and Use Patterns of a Web-Based Intervention for the Prevention of Depression Within a Randomized Controlled Trial
Source: J Med Internet Res. 2013 Aug 20;15(8):e172. doi: 10.2196/jmir.2258 (PMC3757912; doi:10.2196/jmir.2258)
Supplement: Supplementary file 5 [file jmir_v15i8e172_app5.pdf]

CONSORT-EHEALTH Checklist V1.6.2 Report

(based on CONSORT-EHEALTH V1.6), available at [http://tinyurl.com/consort-ehealth-v1-6].

2258

Date completed
2/13/2013 11:24:19
by
Saskia Kelders

Participants, usage and use patterns of a web-based intervention for the prevention of depression

TITLE

1a-i) Identify the mode of delivery in the title
"web-based intervention"
1a-ii) Non-web-based components or important co-interventions in title
none
1a-iii) Primary condition or target group in the title
"for the prevention of depression"

ABSTRACT

1b-i) Key features/functionalities/components of the intervention and comparator in the METHODS section of the ABSTRACT
Yes. The study is about the web-based intervention. The comparators used in the actual RCT-study (different version of the web-based intervention) are not of importance in this study.
1b-ii) Level of human involvement in the METHODS section of the ABSTRACT
"the web-based intervention ‘Living to the full’, a web-based intervention for the prevention of depression employing both a fully automated and human supported format"
1b-iii) Open vs. closed, web-based (self-assessment) vs. face-to-face assessments in the METHODS section of the ABSTRACT
"Demographic and baseline characteristics of participants were collected using an online survey. Log data were collected within the web-based intervention itself."
Recruitment to the intervention is not addressed in the abstract, because it is of less importance to this study than to the actual RCT-manuscript.
1b-iv) RESULTS section in abstract must contain use data
"118 participants fully adhered to the intervention (i.e. started all nine lessons). Participants with an ethnicity other than Dutch were more often adherers ($\chi2$ = 5.470, P = .02) and non-adherers, on average, used the internet more hours per day (F = 3.918, P = .049). A logistic regression showed that being female (B = 0.704; P = .046) and having a higher need for cognition (B = 0.024; P = .02) increased the odds of adhering to the intervention. Overall, participants logged in an average of 4 times per lesson, but adherers logged in significantly more times per lesson than non-adherers (F = 20.710; P < .001). On use patterns, we saw that early non-adherers seem to use less sessions and less time than late non-adherers and adherers; and less sessions to complete the lesson than adherers. Furthermore, late non-adherers seem to have a shorter total duration of sessions than adherers."
1b-v) CONCLUSIONS/DISCUSSION in abstract for negative trials
Not applicable.

INTRODUCTION

2a-i) Problem and the type of system/solution
The goal of the current study is not to invesitgate the effectiveness of this intervention, but rather the usage, adherence and use patterns. The specific information on the goal and rationale of the intervention is given in different papers.
2a-ii) Scientific background, rationale: What is known about the (type of) system
Research into usage of different web-based interventions is discussed. E.g.: "There has been research into the usage and use patterns of web-based interventions. Descriptive studies of freely accessible interventions have shown that they attract a considerable number of visitors, but that these visitors often interact with or access a fraction of what is possible in the intervention [22-30]. Furthermore, many studies have found that increased usage of particular features, such as completing assessments and self-monitoring, increased the effectiveness of the intervention [22, 24, 25, 28-31]. However, insight into the way individuals use an intervention is still lacking."

METHODS

3a) CONSORT: Description of trial design (such as parallel, factorial) including allocation ratio
"The aims of the current study are (1) describe the characteristics of userparticipants and investigate their relationship with adherence; (2) investigate the utilization of the different features of the intervention and possible differences between adherers and non-adherers; (3) identify what use patterns emerge and whether there are differences between adherers and non-adherers."
3b) CONSORT: Important changes to methods after trial commencement (such as eligibility criteria), with reasons
See 3bi
3b-i) Bug fixes, Downtimes, Content Changes
"During the study, no changes were made to the web-based intervention apart from fixing minor bugs."
4a) CONSORT: Eligibility criteria for participants
See below
4a-i) Computer / Internet literacy
"Participants were adults with mild to moderate depressive symptoms (> 9 and < 39 on the Center of Epidemiological Studies – depression scale; CES-D [37]) who completed our online screening procedure."
4a-ii) Open vs. closed, web-based vs. face-to-face assessments:
More detailed information is in the paper about the actual RCT. In this manuscript the following information is given in Multimedia Appendix 1: "Participants were recruited through advertisements in Dutch newspapers between February and March 2011."
4a-iii) Information giving during recruitment
This information will be available with the full RCT study.
4b) CONSORT: Settings and locations where the data were collected
See below
4b-i) Report if outcomes were (self-)assessed through online questionnaires
"Characteristics of participants were collected at baseline using an online questionnaire. "
"Usage of the web-based intervention was measured objectively by log files. "
4b-ii) Report how institutional affiliations are displayed
Not relevant: Potential participants did see that the University of Twente was involved in the study and the intervention, but we feel this does not bias the results.

5) CONSORT: Describe the interventions for each group with sufficient details to allow replication, including how and when they were actually administered

5-i) Mention names, credential, affiliations of the developers, sponsors, and owners

"The authors have been involved in the development of the web-based intervention “Living to the full”."

5-ii) Describe the history/development process

"The intervention was developed employing methods from the CeHRes Roadmap for eHealth development [10] and this process is described in a different paper [46]. "

5-iii) Revisions and updating

"During the study, no changes were made to the web-based intervention apart from fixing minor bugs."

5-iv) Quality assurance methods

Not applicable.

5-v) Ensure replicability by publishing the source code, and/or providing screenshots/screen-capture video, and/or providing flowcharts of the algorithms used

"When logging on to the web-based intervention, participants started in their ‘cockpit’ (Figure 1)"

"A detailed description can be found in Multimedia Appendix 2 and the foundations of these components can be found the parent study [36]."

Detailed descriptions including screenshots have been provided.

5-vi) Digital preservation

See previous subitem: screenshots have been provided.

5-vii) Access

" Participants could access the web-based intervention at any time, from any place, free of charge. "

5-viii) Mode of delivery, features/functionalities/components of the intervention and comparator, and the theoretical framework

A large part of the methods section describes the different features. Moreover, a detailed description is given in multimedia appendix 2.

5-ix) Describe use parameters

"Participants were instructed to complete one lesson per week, but had twelve weeks in total to complete the nine lessons. Participants were free to choose whether they worked through a lesson in one session or in multiple sessions. It was estimated that participants would spend an average of three hours per week on the intervention (online and offline activities combined). "

5-x) Clarify the level of human involvement

"The source of support was either human or automated. To isolate the effect of the source of support, both conditions were designed as comparable as possible regarding length of feedback messages, tailored content and presentation (including a picture of the counselor). To maintain the unique differences between human and automated support (increased possibility for interaction in human support and the increased possibility for timely feedback in automated support), participants in the human support condition had the opportunity to ask questions to their counselor, and participants in the automated support condition received one additional web-based instant feedback message per lesson."

5-xi) Report any prompts/reminders used

"Web-based interaction with the system consisted of doing online exercises, using multimedia content and using personalized features. Interaction in the form of feedback messages (human or automated) was provided within the system as well. Furthermore, interaction with the system took place through automated email messages which were sent to the participants’ email address to remind them to start, continue or complete a lesson. For participants who signed up for text message coaching (see following paragraph), interaction also took place via their mobile phone. This interaction was one-directional; there was no possibility to reply. Furthermore, all participants had the opportunity to contact the research staff by telephone although this possibility was hardly used (a total of approximately five phone calls during the intervention period)."

5-xii) Describe any co-interventions (incl. training/support)

None provided (except for the 'help' feature mentioned in the manuscript).

6a) CONSORT: Completely defined pre-specified primary and secondary outcome measures, including how and when they were assessed

See below

6a-i) Online questionnaires: describe if they were validated for online use and apply CHERRIES items to describe how the questionnaires were designed/deployed

"Characteristics of participants were collected at baseline using an online questionnaire. Depressive symptoms were measured with the CES-D (20 items, score 0-60; higher scores mean more depressive symptoms [37, 47]), anxiety symptoms were measured with the HADS-A (7 items, score 0-21; higher scores mean more anxiety symptoms [48, 49]). Need for cognition was measured using the Need for Cognition Short Form (18 items, score -54 – 54; higher scores mean more need for cognition [32]). Need to belong was measured using Need to Belong Scale (10 items, mean score 1 – 7; higher scores mean more need to belong [50]). Internet usage was measured using one item (i.e. “On average, for how many hours do you use the internet per day?”). Internet experience was measured using 10 items of the following format: ”Do you ever use the following Internet applications?”. The 10 items focus on the usage of search engines, webmail, online shopping, online banking, online communities, photo and video websites, (micro)blogs, chat, radio or music websites, and online (health) courses. The score was attained by counting the number of items that were answered with at least ‘once in a while’ (possible range 0 – 10)."

6a-ii) Describe whether and how “use” (including intensity of use/dosage) was defined/measured/monitored

"Usage of the web-based intervention was measured objectively by log files. From these log files, adherence could be extracted. Adherence was defined as a participant starting lesson 9.

The log files contained a record of actions taken by each participant with for each action the following information: unique participant identification number; action type; action specification; time and day. The action types that were logged were: login, logout, start lesson, start mindfulness, download mindfulness, view success story, view feedback message, start video, turn on text message coach, turn off text message coach and view text message. Action specifications were for example the name of the mindfulness exercise started or which text message was viewed.
"

6a-iii) Describe whether, how, and when qualitative feedback from participants was obtained

not apllicable

6b) CONSORT: Any changes to trial outcomes after the trial commenced, with reasons

not applicable

7a) CONSORT: How sample size was determined

7a-i) Describe whether and how expected attrition was taken into account when calculating the sample size

Not applicable for number of participants (part of the larger study). For the analyses of use patterns:
"Analyses of use patterns were performed on 20 arbitrarily selected participants;"
"We chose to do this analysis only for a small subsample of the data, because the focus of this exploratory analysis was on pattern recognition related to use of features of the interventions. Furthermore, the choice was pragmatic, because due to the lack of software to analyze logfiles at that time, all analyses were done by hand. "

7b) CONSORT: When applicable, explanation of any interim analyses and stopping guidelines

Not applicable fot this study on usage.

8a) CONSORT: Method used to generate the random allocation sequence

From the description of the parent study (multimedia appendix 1):

"A total of 239 respondents fulfilled the inclusion criteria, completed the online baseline questionnaire and were automatically randomized to one of eight intervention arms. "

8b) CONSORT: Type of randomisation; details of any restriction (such as blocking and block size)

This information is presented in the parent study.

9) CONSORT: Mechanism used to implement the random allocation sequence (such as sequentially numbered containers), describing any steps taken to conceal the sequence until interventions were assigned

This information is presented in the parent study.

10) CONSORT: Who generated the random allocation sequence, who enrolled participants, and who assigned participants to interventions

This information is presented in the parent study.

11a) CONSORT: Blinding - If done, who was blinded after assignment to interventions (for example, participants, care providers, those assessing outcomes) and how

11a-i) Specify who was blinded, and who wasn't

"Respondents were not blinded to their randomized arm, but had no in-depth knowledge of the other arms."

11a-ii) Discuss e.g., whether participants knew which intervention was the "intervention of interest" and which one was the "comparator"

"Respondents were not blinded to their randomized arm, but had no in-depth knowledge of the other arms."
All versions of the intervention were 'of interest'.

11b) CONSORT: If relevant, description of the similarity of interventions

Not applicable.

12a) CONSORT: Statistical methods used to compare groups for primary and secondary outcomes

"Differences between adherers and non-adherers were investigated using one-way analyses of variance (ANOVA) and χ2 tests. Logistic regression was used to assess whether baseline characteristics predicted adherence"

12a-i) Imputation techniques to deal with attrition / missing values

Not applicable (there was no missing data due to the design of the study).

12b) CONSORT: Methods for additional analyses, such as subgroup analyses and adjusted analyses

"Differences between adherers and non-adherers were investigated using one-way analyses of variance (ANOVA) and χ2 tests. Logistic regression was used to assess whether baseline characteristics predicted adherence"

RESULTS

13a) CONSORT: For each group, the numbers of participants who were randomly assigned, received intended treatment, and were analysed for the primary outcome

"Table 1 – Experimental groups of the fractional factorial design and the number of participants
GroupSupportText messagesExperienceTailoringPersonalizationParticipants (n)
1AutomatedYesHighHighHigh11
2AutomatedYesLowLowLow43
3AutomatedNoHighLowLow36
4AutomatedNoLowHighHigh23
5HumanYesHighLowHigh52
6HumanYesLowHighLow19
7HumanNoHighHighLow35
8HumanNoLowLowHigh20"
(Multimedia Appendix 1)

13b) CONSORT: For each group, losses and exclusions after randomisation, together with reasons

This information is given in the parent study.

13b-i) Attrition diagram

"Table 3. Furthest lesson reached for all participants
Lesson reachedn%cumulative %
1115.3100
2209.794.8
3178.385.1
462.976.8
531.573.9
6146.872.4
794.465.6
883.961.2
911857.357.3"

14a) CONSORT: Dates defining the periods of recruitment and follow-up

There were no follow-up questionnaires that were used in this study. On the period of the intervention:
"Participants were instructed to complete one lesson per week, but had twelve weeks in total to complete the nine lessons. "

14a-i) Indicate if critical "secular events" fell into the study period

Not applicable.

14b) CONSORT: Why the trial ended or was stopped (early)

Not applicable.

15) CONSORT: A table showing baseline demographic and clinical characteristics for each group

"Table 2. Baseline demographics and outcome measures of all participants, adherers and non-adherers"

15-i) Report demographics associated with digital divide issues

Yes, see "Table 2. Baseline demographics and outcome measures of all participants, adherers and non-adherers"

16a) CONSORT: For each group, number of participants (denominator) included in each analysis and whether the analysis was by original assigned groups

16-i) Report multiple "denominators" and provide definitions

Not applicable for this study.

16-ii) Primary analysis should be intent-to-treat

Not applicable for this study.

17a) CONSORT: For each primary and secondary outcome, results for each group, and the estimated effect size and its precision (such as 95% confidence interval)

Not applicable for this study

17a-i) Presentation of process outcomes such as metrics of use and intensity of use

"Table 4. User actions of adherers and non-adherers"
"Table 5. Mean number of sessions and duration for early non-adherers (n = 5), late non-adherers (n = 5) and adherers (n = 10)"

17b) CONSORT: For binary outcomes, presentation of both absolute and relative effect sizes is recommended

Not applicable for this study.

18) CONSORT: Results of any other analyses performed, including subgroup analyses and adjusted analyses, distinguishing pre-specified from exploratory

The paper mainly includes subgroup analyses. It is made clear that this is not a randomized group.

18-i) Subgroup analysis of comparing only users

See previous subitem.

19) CONSORT: All important harms or unintended effects in each group

No unintended effects have been found.

19-i) Include privacy breaches, technical problems

No unintended effects have been found.

19-ii) Include qualitative feedback from participants or observations from staff/researchers

This will be included in the parent study.

DISCUSSION

20) CONSORT: Trial limitations, addressing sources of potential bias, imprecision, multiplicity of analyses

20-i) Typical limitations in ehealth trials

The 'typical' limitations are not applicable because we did not look at the effectiveness of the intervention. However, several limitations have been addressed.

21) CONSORT: Generalisability (external validity, applicability) of the trial findings

21-i) Generalizability to other populations

"Another limitation is the issue of generalizability. Our study was done on the data of one intervention for the prevention of depression, which has been used by mainly higher educated Dutch women. Furthermore, we only investigated the use patterns of a small sample of these participants. The observed use patterns may be specific for this group using this intervention. However, many interventions, especially mental health interventions, have similar characteristics [16] and reach the same audience as stated earlier. "

21-ii) Discuss if there were elements in the RCT that would be different in a routine application setting

This will be addressed in the parent study.

22) CONSORT: Interpretation consistent with results, balancing benefits and harms, and considering other relevant evidence

22-i) Restate study questions and summarize the answers suggested by the data, starting with primary outcomes and process outcomes (use)

"The aims of this study were (1) describe the characteristics of participants and investigate their relationship with adherence; (2) investigate the utilization of the different features of the intervention and possible differences between adherers and non-adherers; (3) identify what use patterns emerge and whether there are differences between adherers and non-adherers. Below, the results regarding these aims will be discussed."

22-ii) Highlight unanswered new questions, suggest future research

Several future research topics are suggested. e.g.:
"These two findings show a need to further investigate the role of support and feedback in web-based interventions. "

Other information

23) CONSORT: Registration number and name of trial registry

"Trial Registration: Dutch Trial Register NTR3007"

24) CONSORT: Where the full trial protocol can be accessed, if available

"Trial Registration: Dutch Trial Register NTR3007"

25) CONSORT: Sources of funding and other support (such as supply of drugs), role of funders

"The authors have been involved in the development of the web-based intervention "Living to the full"."

X26-i) Comment on ethics committee approval

"The study was approved by an independent medical ethics committee (METIGG; no. NL33619.097.10) and recorded in the Dutch primary trial register for clinical trials (NTR3007)." (Multimedia Appendix 1)

x26-ii) Outline informed consent procedures

" After viewing on screen information on the study and having the opportunity to download this information, informed consent was obtained from the participant through a checkbox and a pop-up screen to check whether they were sure to give informed consent. " (Multimedia Appendix 1)

X26-iii) Safety and security procedures

"Participants had no contact with the research staff, apart from the ability to ask questions via email or telephone." (Multimedia Appendix 1).

X27-i) State the relation of the study team towards the system being evaluated

"The authors have been involved in the development of the web-based intervention "Living to the full"."
